# Engineering State‐of‐the‐Art Plasmonic Nanomaterials for SERS‐Based Clinical Liquid Biopsy Applications

**DOI:** 10.1002/advs.201900730

**Published:** 2019-09-30

**Authors:** Jing Wang, Kevin M. Koo, Yuling Wang, Matt Trau

**Affiliations:** ^1^ Centre for Personalized Nanomedicine Australian Institute for Bioengineering and Nanotechnology (AIBN) The University of Queensland Brisbane QLD 4072 Australia; ^2^ Department of Molecular Sciences ARC Excellence Centre for Nanoscale BioPhotonics Faculty of Science and Engineering Macquarie University Sydney NSW 2109 Australia; ^3^ School of Chemistry and Molecular Biosciences The University of Queensland Brisbane QLD 4072 Australia

**Keywords:** clinical translation, liquid biopsies, plasmonic nanomaterials, precision oncology, surface‐enhanced Raman scattering

## Abstract

Precision oncology, defined as the use of the molecular understanding of cancer to implement personalized patient treatment, is currently at the heart of revolutionizing oncology practice. Due to the need for repeated molecular tumor analyses in facilitating precision oncology, liquid biopsies, which involve the detection of noninvasive cancer biomarkers in circulation, may be a critical key. Yet, existing liquid biopsy analysis technologies are still undergoing an evolution to address the challenges of analyzing trace quantities of circulating tumor biomarkers reliably and cost effectively. Consequently, the recent emergence of cutting‐edge plasmonic nanomaterials represents a paradigm shift in harnessing the unique merits of surface‐enhanced Raman scattering (SERS) biosensing platforms for clinical liquid biopsy applications. Herein, an expansive review on the design/synthesis of a new generation of diverse plasmonic nanomaterials, and an updated evaluation of their demonstrated SERS‐based uses in liquid biopsies, such as circulating tumor cells, tumor‐derived extracellular vesicles, as well as circulating cancer proteins, and tumor nucleic acids is presented. Existing challenges impeding the clinical translation of plasmonic nanomaterials for SERS‐based liquid biopsy applications are also identified, and outlooks and insights into advancing this rapidly growing field for practical patient use are provided.

## Graphical abstract

**In a bid to facilitate next‐generation plasmonic nanomaterials for clinical exploration of SERS‐based liquid biopsy** applications, a detailed overview of up‐to‐date plasmonic nanomaterials that are designed for SERS applications and newly developed SERS technologies for liquid biopsies, is provided.

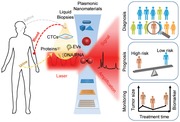

## 1 Introduction

Precision oncology is an ongoing revolution in cancer management that involves the use of tumor molecular profiling to identify actionable cancer driver mutations in individual patients for tailored cancer treatments. As compared to the existing “one‐size‐fits‐all” treatment strategy across all patients, the potential benefits of precision oncology include improved treatment quality from targeted therapeutics, and decreased costings through reduction of unnecessary diagnostic tests and therapies. Therefore, for successful administration of precision oncology, a convenient noninvasive biopsy approach which can provide comprehensive tumor molecular information is greatly required. In this regard, the use of liquid biopsies has begun to showcase its clinical promise in precision oncology.

Liquid biopsies involve the minimally invasive sampling of circulating bodily fluids (such as blood, urine, or sputum) for detection of cancer biomarkers such as circulating tumor cells (CTCs), tumor‐derived extracellular vesicles (EVs), as well as circulating cancer proteins and tumor nucleic acids (NAs).^1^ As compared to tissue‐based biopsies from limited tumor regions, detection and characterization of such circulating biomarkers could provide more comprehensive molecular information on the primary and metastatic tumors present in a patient.[qv: 1b] Many recent studies have illustrated the clinical potential of using liquid biopsies for diagnosing early‐stage cancer, monitoring tumor progression, tracking treatment responses, and assessing residual disease.^1^, ^2^ Additionally, as liquid biopsies can be obtained in an easily accessible and minimally invasive way, it could be used to determine and follow the dynamic molecular makeup of a patient's tumor longitudinally. Therefore, liquid biopsies may be a stepping stone in overcoming the longstanding cancer detection challenges related to tumor heterogeneity and ultimately promote precision oncology.

Due to limited trace amounts in biofluids, the analysis of cancer biomarkers in liquid biopsies is a daunting task. To date, liquid biopsy specimens can be analyzed for biomarkers using either protein (e.g., western blot, enzyme‐linked immunosorbent assay (ELISA), or fluorescence‐assisted cell sorting) or NA (e.g., polymerase chain reaction (PCR)‐based or next‐generation sequencing (NGS)) methods. While this repertoire of analysis techniques has undoubtedly enabled great advancements for liquid biopsy applications, their performance in terms of detection sensitivity, multiplexing capacity, and assay cost is still to be improved further for the potential of liquid biopsies in clinical cancer detection and treatment response prediction/monitoring. In this respect, modern innovative research into nanomaterials has led to unprecedented detection capabilities of analytical techniques, which may well be harnessed for advancing liquid biopsy applications. A prime example is the engineering of cutting‐edge plasmonic nanomaterials for surface‐enhanced Raman scattering (SERS) analyses.

**Figure 1:**
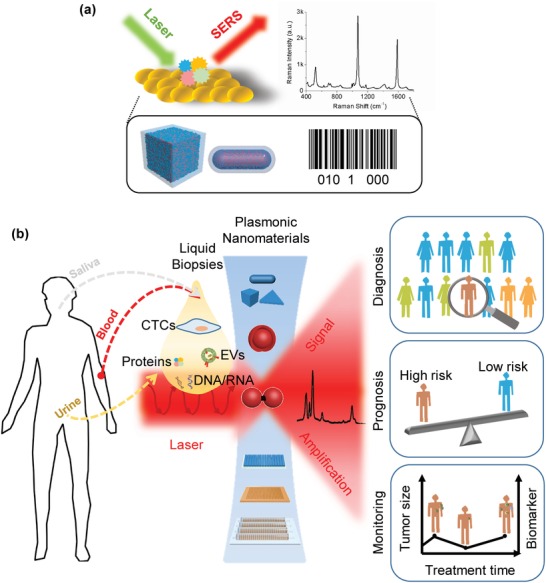
a) Enhanced Raman scattering of molecules on or near the surface of plasmonic nanomaterials provides fingerprint information of molecules, and could be used as a barcode signal in SERS measurements. b) Liquid biopsy could provide comprehensive molecular information on the primary and metastatic tumors present in a patient. The development of high‐performance plasmonic nanomaterials further amplifies SERS signals for broad potential clinical applications.

SERS is a powerful analytical technique that has shown promise for ultrasensitive liquid biopsy analyses. Essentially, SERS is described as the amplified inelastic Raman scattering of molecules on or near the SERS‐active substrates (most often plasmonic metallic nanomaterials), as shown in **Figure** **1**a. Fundamentally, the SERS signal is generated due to two different mechanisms: chemical mechanism and electromagnetic mechanism (EM). The chemical mechanism refers to the electronic interaction between substrates and adsorbed molecules,^3^ which depends on the binding affinity between molecules and substrates and provides the minor enhancement magnitude (10^2^–10^3^).^4^ EM provides the contribution through enhancing the electromagnetic field around plasmonic structures generated upon the excitation of a localized surface plasmon resonance (LSPR) by the incident light. Plasmonic nanomaterials are such nanomaterials which conduction electrons can be coherently excited by electromagnetic radiation of incident light to oscillate collectively at metal/dielectric interfaces. The maximum SERS enhancement factor (EF) arising from EM contribution on plasmonic nanomaterials is in the order of 10^10^–10^11^,^5^ which is sufficient for the detection of single molecules.^6^ Raman scattering signals of molecules on the surface of plasmonic nanomaterials are thus amplified mainly due to the LSPR effect.

SERS can be used to directly characterize the fingerprint information of native targets adsorbed close to the surface of plasmonic nanomaterials, or for selective target detection via encoded labels (termed as “SERS nanotags”) that have surface functionalization of organic Raman reporter molecules and/or target‐specific binding molecules. In particular, SERS nanotags possess multiple merits, such as single‐particle sensing activity,^7^ multiplexing capacity,^8^ and photostability;[qv: 8b] thus rendering the SERS technique promising for diverse biosensing applications in place of conventional optical labels (e.g., fluorescent dyes and quantum dots). Currently, a wide range of state‐of‐the‐art plasmonic nanomaterials has been conceived to further progress the SERS enhancement efficiency for unparalleled detection sensitivity, and reproducible signal readouts of exceptional uniformity and stability.

In order for plasmonic nanomaterials to effectively contribute toward SERS‐based clinical liquid biopsy applications, it is of our opinion that the clinical usage feasibility of advanced plasmonic nanomaterials is as equally important as their design and synthesis. Despite numerous excellent reviews on plasmonic nanostructures for proof‐of‐concept SERS biomedical applications in recent times,^5^, ^9^ a comprehensive focus on plasmonic nanomaterials and their clinical translation for SERS‐based liquid biopsy applications such as cancer diagnosis, risk prediction, and treatment monitoring has yet to be summarized. In a bid to address this crucial point, we provide herein an extensive review on the rational design/synthesis of state‐of‐the‐art plasmonic nanomaterials as SERS substrates, and up‐to‐date progress (within last 3 years) of plasmonic nanomaterial‐based SERS analyses which have been applied for liquid biopsies (Figure 1b). We also identify existing challenges impeding the translation of engineered plasmonic nanomaterials for clinical SERS‐based biosensing, and share our perspectives and insights on how to advance this rapidly evolving field.

## 2 Engineering Cutting‐Edge Plasmonic Nanomaterials for SERS

Plasmonic nanomaterials have been applied as the signal amplification substrates for SERS sensing due to the excellent enhancement efficiency (an average electromagnetic enhancement of ≈10^6^)^10^ and as drug containers for SERS nanotag‐based therapy.^11^ Colloidal gold and silver nanospheres are the most commonly used plasmonic nanomaterials for SERS‐based biosensing due to their simple synthesis and easy functionalization. However, the tunable LSPR ranges of gold and silver nanospheres are narrow^12^ and not in the near‐infrared (NIR) region from 650 to 900 nm (ideal for biomedical applications due to the low light attenuation and autofluorescence from biomolecules).^13^ Additionally, a single gold nanosphere can only generate an EF on the order of 10^2^–10^3^.^14^ In contrast, dimers or oligomers generate higher enhancement efficiency than single nanospheres because of the intensified electromagnetic enhancement due to interparticle LSPR coupling effects and increased absorbance in the NIR region owing to a redshift of the LSPR.

The SERS signal enhancement ability of plasmonic nanomaterials mainly relies on the LSPR coupling effect which is strongly dependent on the size,^15^ shape,^15^, ^16^ chemical composition,^17^ local dielectric environment,^18^ and interparticle interaction.^19^ Generally, strong electromagnetic fields (termed as “hot spots”) could be created by sharp tips,^20^ vertices,^21^ nanoporous frameworks in nanostructures,^22^ intra‐nanogaps within nanostructures,^23^ and crevices or inter‐nanogaps between nanostructures.^24^ The enhancement efficiency of a hot spot is affected by many factors, such as curvature degree, gap size between nanostructures, and laser polarization.^25^ Recent progress has produced high‐performance plasmonic nanomaterials with excellent uniformity, reproducibility, and stability. In this section, we will summarize the design and synthesis of different structures of plasmonic nanomaterials as modern SERS substrates. Plasmonic nanomaterials that are highly promising or have already been demonstrated for liquid biopsy applications are further highlighted for discussion.

### 2.1 Anisotropic Plasmonic Nanoparticles

Anisotropic particles have nonspherical structures and provide strong SERS enhancements by moving the LSPR band within the NIR region and creating “built‐in” hot spots at locations of tips, vertices, or edges. Typical anisotropic structures include nanorods,^26^ nanotriangles,^27^ nanocubes,^21^, ^28^ polyhedral nanoparticles,^29^ nanoflowers,^30^ nanostars,^20^ highly branched nanocrystals,^31^ concave nanocrystals,^32^ cubic nanoframes,^32^ and microcages.^33^ These anisotropic particles are attractive for biological and biomedical applications due to their capability of providing single‐particle SERS activity.

#### 2.1.1 Template‐Based Synthesis

**Figure 2:**
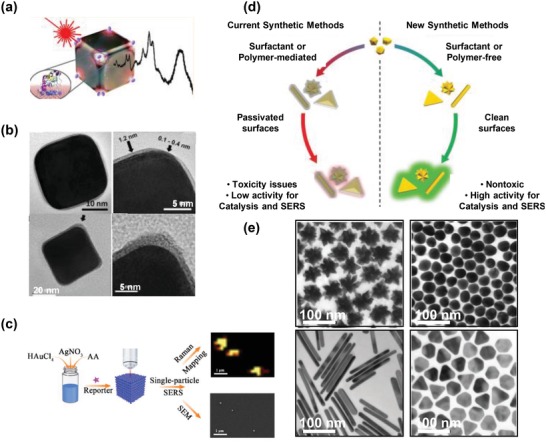
a) Representation of contacts between proteins and corner sites of silver nanocubes. b) Bright‐field transmission electron microscopy (TEM) images of a single silver nanocube before (top) and after (bottom) incubation with cytochrome c; images on the right are the corresponding magnification of corners. Reproduced with permission.^21^ Copyright 2017, American Chemical Society. c) One‐pot synthesis process of nanodot‐decorated gold–silver alloy nanoboxes with single‐particle SERS activity. Reproduced with permission.^38^ Copyright 2018, American Chemical Society. d) Comparison of shape‐controlled syntheses using surface blocking reagents (conventional synthetic methods), and in absence of surface blocking reagents. e) TEM images of nanostars, nanospheres, nanorods, and nanoplates synthesized without using surface blocking reagents. Reproduced with permission.^40^ Copyright 2017, Wiley‐VCH.

Surfactant‐ and polymer‐mediated syntheses remain the most common methods to generate anisotropic particles. Matteini et al. utilized polyvinylpyrrolidone (PVP) as a structure‐directing polymer to synthesize silver nanocubes for the detection of proteins in aqueous media at physiological pH (**Figure** **2**a).^21^ In this work, the synthesis of silver nanocubes relied on the PVP's stronger binding affinity to silver facets (100) with respect to (111) facets. The synthesized silver nanocubes resulted in an incomplete and patchy monolayer (thickness of 0.3–0.5 nm) at corners and multiple layers (thickness of 1.1–1.8 nm) on cube faces (Figure 2b, top). Such PVP coatings drove the preferential interaction of proteins with the corners of silver nanocubes by shielding the contacts between proteins and silver nanocube faces (Figure 2b, bottom). A limit of detection (LOD) of ≈(5–10) × 10^−9^ m (equal to ≈60–120 ng mL^−1^) cytochrome c (12 kD) was achieved by such site‐selective gathering of protein molecules at the hot spots generated on the corners of individual silver nanocubes.^21^ However, this detection sensitivity is not superior to ELISA (≈pg mL^−1^–ng mL^−1^), and this nanostructure might not be suitable for big‐sized biomarker (>10 nm) analysis given the limited hot spot area at corners.

Generally, while surfactants or polymers contribute to the precise control of nanoparticle morphology, the use of these structure‐directing agents might i) weaken SERS performance due to the occupied surfaces or increased distances to surfaces of anisotropic particles,^32^, ^34^ ii) generate detectable background signals which can interfere with SERS signals of target molecules,^35^ and iii) cause apparent cytotoxicity and affect downstream molecular analyses (e.g., PCR analysis after SERS characterization of CTCs and EVs) resulting from the desorption or residual molecules in solution.^36^ Although stubborn capping agents could be removed via washing strategies, there will always be residuals which are resistant to removal.^37^

#### 2.1.2 Template‐Free Synthesis

Given the drawbacks of using structure‐directing agents for the synthesis of anisotropic nanoparticles, template‐free synthesis methods are being developed. Li et al. synthesized template‐free nanodot‐decorated gold–silver alloy nanoboxes with single‐particle SERS activity (Figure 2c)^38^ for the detection of circulating soluble cancer protein biomarkers.^39^ Ascorbic acid was solely used for the reduction of hydrogen tetrachloroaurate trihydrate and silver nitrate, and was performed in a one‐pot synthesis in aqueous phase under ambient temperature. Meanwhile, Wall et al. proposed a simple, green chemistry, and universal system for synthesizing nanostars, nanospheres, nanorods, and nanoplates (Figure 2d,e).^40^ The authors demonstrated that these synthesized template‐free nanoparticles have better SERS performance as compared to analogous nanoparticles with surfactant or polymer coatings. Such template‐free synthesis methods could minimize contamination and/or cytotoxicity for CTCs or EVs, which are particularly useful for ex vivo culturing of CTCs and EVs for functional evaluation.

### 2.2 Nanostructures with Intra‐Nanogaps

Apart from enhancing SERS signals by creating sharp tips and vertices in single anisotropic particles, great efforts have been made to generate hot spots through building nanogaps within nanostructures. The size and morphology of nanogaps pose a significant effect on the plasmon mode and signal intensity. Generally, the electromagnetic field intensity strengthens with the decrease of nanogap sizes and the maximum SERS intensity is achieved at ≈1 nm region.^41^

#### 2.2.1 Accessible Intra‐Nanogaps

**Figure 3:**
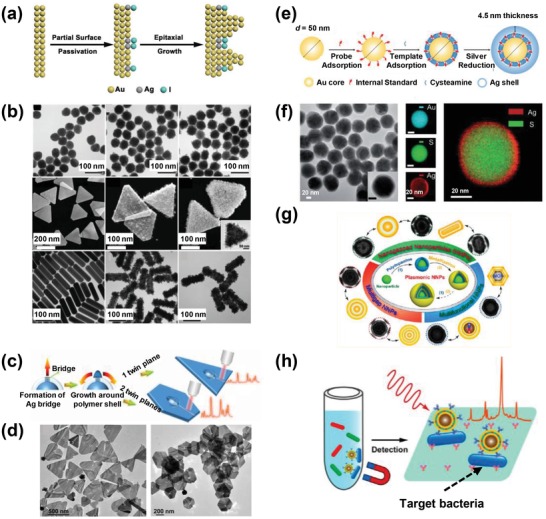
a) Formation of islands‐on‐gold nanostructures. b) TEM images of gold nanospheres and islands‐on‐gold nanospheres (top row), gold nanoplates and islands‐on‐gold nanoplates (middle row), and gold nanorods and islands‐on‐gold nanorods (bottom row) with low (middle column) to high (right column) densities of nanoislands. Scale bar of the inset is 50 nm. Reproduced with permission.^42^ Copyright 2018, Wiley‐VCH. c) Schematic illustration and d) TEM images of triangular and hexagonal silver nanoplates with nanogaps. Reproduced with permission.^43^ Copyright 2018, American Chemical Society. e) Synthesis of core–shell nanoparticles with embedded molecules. f) Corresponding TEM and scanning TEM images based on gold, sulfur, silver, and sulfur/silver signals. Reproduced with permission.^45^ Copyright 2015, Wiley‐VCH. g) Synthesis of versatile plasmonic nanogapped nanoparticles based on polydopamine coatings. h) Schematic of the immunoassay of using SERS nanotags with magnetic cores for bacteria detection. Reproduced with permission.[qv: 23d] Copyright 2016, American Chemical Society.

To produce abundantly accessible nanogaps, Fan et al. reported a convenient, universal method that generally constructed well‐defined gold–silver alloy nanoislands on the surface of pre‐existing PVP‐stabilized gold seeds (**Figure** **3**a,b).^42^ The nanogap distance between nanoislands or the density of nanoislands could be tuned by varying the concentration of chloroauric acid. The SERS signals increased with the density of nanoislands, and the maximum single‐particle SERS EF was estimated to be ≈5 × 10^6^. The key to the synthesis is utilizing a partial surface passivation strategy to shift the crystal growth mode from “Frank–van der Merwe” mode (layer‐by‐layer growth) to “Volmer–Weber” mode (islands growth) based on the strong adsorption of iodide on silver‐coated gold seed surfaces. This method directly builds secondary nanostructures on pre‐existing plasmonic nanocrystals during the initial synthesis, which is more efficient and reproducible than methods that engineer the presynthesized colloidal nanoparticles into secondary structures. Additionally, the described nanoislands have also been successfully formed on gold nanospheres, nanoplates, and nanorods (Figure 3b).

To create a nanogap with a large accessible area, Jiang et al. constructed a silver nanoplate with a long, ultranarrow gap (90 nm in length and 2 nm in width) (Figure 3c,d).^43^ Silver nanoparticles were first encapsulated in polystyrene‐*block*‐poly(acrylic acid) and served as growth seeds. The silver domain then extended from one point of the encapsulated seed but did not merge at the meet‐up point, leaving a long narrow gap. This gapped nanoplate showed 2–4 times stronger signals than its gap‐absent counterpart, and has demonstrated its capability in detecting down to 10^−9^ m of 2‐naphthalenethiol due to the large area of hot spots. Nevertheless, the width of 2 nm might still be too narrow for bigger biomolecules (e.g., bovine serum albumin, ≥3 nm) to enter into the nanogap.^44^ As such, to be applicable for liquid biopsy analyses, further structural modification of this gapped nanomaterial is required.

#### 2.2.2 Enclosed Intra‐Nanogaps

Core–shell gapped nanoparticles with a built‐in dielectric gap separating core and shell have emerged as an attractive type of SERS nanotags for liquid biopsy analyses due to strong, stable SERS signals. The SERS signals of Raman reporters within the nanogap (≤10 nm) can be enormously amplified because of the enhanced electromagnetic field. The shell structure can also prevent Raman reporters from contaminant‐induced desorption and enzymatic degradation, as well as signal variations caused by microenvironmental interferences. These two merits make core–shell‐gapped nanoparticles competent for quantitative SERS analyses. Common practices to generate controllable, uniform interior nanogaps mainly use DNA,[qv: 23a] small molecules,[qv: 23c,45] and polymers[qv: 23d] as dielectric spacers. For example, Shen et al. constructed gapped SERS nanotags with two types of small molecules in the dielectric layer, including framework molecules (i.e., cysteamine) to form the shell and Raman reporter molecules as a Raman internal standard (Figure 3e,f).^45^ The introduction of framework molecules provides the versatility to encode SERS nanotags with different Raman reporters. Such gapped SERS nanotags have also demonstrated their vital roles in the direct detection of target molecules with much improved dynamic range, reproducibility, and reliability.^45^ It is therefore ideal to use SERS nanotags with enclosed gaps for the quantification of liquid biopsy biomarkers.

Zhou et al. used polydopamine as a nanoscale spacer to provide great flexibility in tailoring structures and properties of core–shell nanoparticles with built‐in nanogaps (Figure 3g).[qv: 23d] The universal adhesion of polydopamine on diverse colloidal substrates allows for the customized synthesis of multishell gapped nanoparticles and multifunctional particles with different cores, such as SERS nanotags with magnetic cores. As a proof‐of‐concept, they have demonstrated that bioconjugated SERS nanotags with magnetic cores were capable of achieving efficient magnetic separation, ultrasensitive Raman detection, and effective photothermal killing of a common food‐borne pathogen, *Escherichia coli. O157: H7* (Figure 3h). Similar multifunctional plasmonic core–shell nanoparticles have been applied for simultaneous isolation and characterization of specific CTC subpopulations in whole blood.^46^ Compared to conventional two‐step CTC analyses (i.e., immunomagnetic isolation and labeling), the use of such nanomaterials might simplify and accelerate the CTC analysis process.

### 2.3 Nanoassemblies with Interparticle Nanogaps

Nanoassemblies with interparticle nanogaps are of special interest due to their ultrastrong electromagnetic fields and controllable interparticle distance. However, these promising nanoassemblies have shown limited practical applications due to the lack of design principles and low yield of target nanoassemblies.^41^

#### 2.3.1 Thiolated Linker–Constructed Assembly

**Figure 4:**
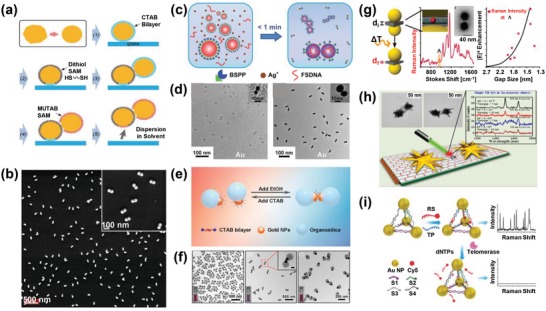
a) Schematic representation of substrate‐based sequential dimer assembling. b) SEM image of ideal dimers formed on a glass substrate. Reproduced with permission.[qv: 24f] Copyright 2017, Wiley‐VCH. c) Representation of the silver‐ion soldering process for forming particle assemblies. d) TEM images of gel‐isolated gold nanoparticle dimers of 5.5 and 13.5 nm, showing 95% and 98% yields, respectively. Reproduced with permission.^48^ Copyright 2016, Royal Society of Chemistry. e) Reversible self‐assembly of Janus gold nanoparticle dimers. f) TEM images of original (left), self‐assembled (middle), and disassembled (right) Janus gold nanoparticles. Scale bar of the inset is 50 nm. Reproduced with permission.^49^ Copyright 2016, American Chemical Society. g) Schematic design of gold nanoparticle dimers on DNA origami. Reproduced with permission.[qv: 50c] Copyright 2016, American Chemical Society. h) DNA origami gold nanostar dimers. Reproduced with permission.[qv: 24b] Copyright 2017, American Chemical Society. i) Schematic illustration of gold pyramid‐based telomerase detection. Reproduced with permission.[qv: 24g] Copyright 2016, Wiley‐VCH.

The interparticle distance in nanoassemblies has a significant effect on the plasmon mode and signal intensity; thiolated linkers have thus been proposed to control the nanoassemblies through forming distance‐adjustable nanogaps. For example, Guarrotxena and Bazan synthesizd silver nanoassemblies using thiolated reporter linkers. After further surface functionalization with target‐specific antibodies, these silver nanoassemblies have been used for the detection of human α‐thrombin, myoglobin, and c‐reactive protein.^47^ However, for solution‐based synthetic methods, the use of thiolated linkers often leads to uncontrollable aggregation and heterogeneous size distributions of nanoassemblies, which in turn reduces the reproducibility of SERS measurements. To avoid over‐crosslinking of nanoparticles into bulky precipitates and to improve the purity of nanoassemblies, Yoon et al. proposed a substrate‐based sequential dimer assembling process (**Figure** **4**a).[qv: 24f] Monomeric gold nanoparticles were first electrostatically immobilized onto a bare glass substrate and then covalently conjugated with second nanoparticles via dithiol linkers. The resulting assemblies with 87% of dimers (Figure 4b) were then collected by detachment from the glass surface. Notwithstanding, this method is time‐consuming and requires multiple steps, including two conjugation and one detachment steps. Therefore, to promote the applications of nanoassemblies as SERS nanotags for liquid biopsy biomarker barcoding, it is of great demand to develop a method that can efficiently generate high‐purity target assemblies as well as synthesize new Raman reporters with dithiol groups.

#### 2.3.2 Thiolated Linker–Free Assembly

To address the limitations of using thiolated linkers for building nanoassemblies, thiolated linker–free strategies have been devised. Liu et al. proposed a solution‐based silver ion soldering process for forming nanoassemblies (Figure 4c).^48^ The addition of silver ions stripped off of bis(*p*‐sulfonatophenyl) phenyl phosphine (BSPP) from nanoparticle surfaces based on the strong affinity between silver ions and BSPP, and subsequently led to the destabilization of BSPP‐stabilized gold assembly. The silver ion–triggered aggregation process reached a steady self‐limiting phase immediately upon adding a certain amount of FSDNA (mechanically shortened fish sperm DNA). FSDNA adsorbed onto the partially exposed nanoparticle surfaces and terminated further clusterization by increasing steric repulsion. The target assemblies (e.g., dimers) were then purified through high‐resolution gel isolation, showing high purity (≥91%) of dimers (Figure 4d). Compared to thiolated linker–based strategies, this synthesis method avoids the over‐crosslinking of nanoparticles into bulky precipitates and generates dimers with excellent stability, high purity, and minimum surface passivation. Nonetheless, a purification process is still needed to yield relatively pure target nanostructures, making this synthetic method unsuitable for mass production.

Hu et al. reported a strategy to generate precisely controllable particle assemblies by tuning interparticle forces and steric hindrance, without any purification process (Figure 4e).^49^ Hexadecyltrimethylammonium bromide (CTAB)‐coated Janus gold nanoparticles were used as building blocks, and for manipulating the direction of van der Waals forces to induce directional binding of nanoparticles in ethanol. The proportion of dimers generated by this method approached ≈70% (Figure 4f), and the SERS EF of corresponding dimers reached ≈10^6^. However, the use of toxic chemical CTAB will limit practical biosensing usage, particularly for in vivo biological applications.

An alternative thiolated linker–free method is DNA origami–based nanoparticle assembly strategies.[qv: 24b,50] DNA origami–based methods utilized DNA origamis with docking sites to precisely control the location of nanoparticles and to create ≤10 nm nanogaps between nanoparticles. The nanogap distance is controllable[qv: 24b,50c] and can be narrowed down to 1–2 nm by the optothermal‐induced shrinking of a DNA origami to generate strong SERS enhancement (Figure 4g).[qv: 50c] The yield of target assemblies (i.e., particle dimers) reached as high as 72%.[qv: 50a] In addition, the site‐specific placement of analytes at hot spot regions could be achieved with DNA staples,[qv: 24b,50c] which not only maximized SERS signals but also minimized signal variations caused by varied positioning of analytes. As such, DNA origami–based particle assembly methods have demonstrated outstanding merits, including excellent nanogap‐induced SERS enhancement, high yield of target assemblies, precise placement of analytes at hot spot regions, and minimum cytotoxicity.

Most of DNA origami–based particle assembly methods have only been applied for the dye molecule detection due to limited accessible nanogaps (≤10 nm). To provide relatively bigger nanogaps for the attachment of biomolecules while still ensuring excellent SERS enhancement, Tanwar et al. immobilized gold nanostars on DNA origami substrates to create DNA origami–based dimeric gold nanostars with nanogaps of 7 and 13 nm, which generated SERS EFs of 2 × 10^10^ and 8 × 10^9^, respectively (Figure 4h).[qv: 24b] The resultant EF is at least 100‐fold higher than dimeric spherical gold nanoparticles (≈10^7^). Currently, the feasibility of using DNA origami–based dimeric gold nanostars for SERS measurements of biomolecules has yet to be evaluated, and the potential interference of DNA background signals in such measurements needs to be further studied.

Similar to the DNA origami–based method, Xu et al. proposed strategies using DNA‐frame‐based silver or gold pyramids for disease biomarker detection.[qv: 24d,g] The yield of ≈86% nanoparticle pyramids was achieved.[qv: 24g] Using the detection of telomerase as an example, telomerase primers and dye molecules were inserted into DNA frames for target identification and signal transduction, respectively (Figure 4i).[qv: 24g] In the presence of telomerase and deoxynucleotides, telomerase triggered the strand extension of telomerase primers, which resulted in the replacement of dye molecules and subsequently a reduced SERS signal level. Such DNA‐frame‐based nanoparticle pyramids enabled an ultrasensitive detection of telomerase with an LOD of 6.2 × 10^−15^ IU. Given the excellent detection sensitivity and multiplexity provided by DNA‐frame‐based nanoparticle pyramids, it might be interesting to explore their feasibility in detecting alternative types of liquid biopsy biomarkers, such as circulating tumor NAs.

### 2.4 Supercrystals

Plasmonic supercrystals that are formed by self‐assembling individual nanoparticles into compact, well‐defined 3D structures, possess intrinsic characteristics of individual nanoparticles as well as unique collective optical and electronic properties. Particularly, plasmonic supercrystals provide tunable plasmon resonances and intensified electromagnetic fields at nanoparticle interstices. Bian et al. used a binary solvent diffusion method to synthesize self‐assembled hexagonal close‐packed 3D gold nanoparticle supercrystals.^51^ The growth of gold nanoparticle supercrystals was achieved by slowly driving the nanoparticle solution to supersaturation with increasing antisolvent concentrations. The size and quality of gold nanoparticle supercrystals were tuned by adjusting the initial nanoparticle concentration and the diffusion speed.

**Figure 5:**
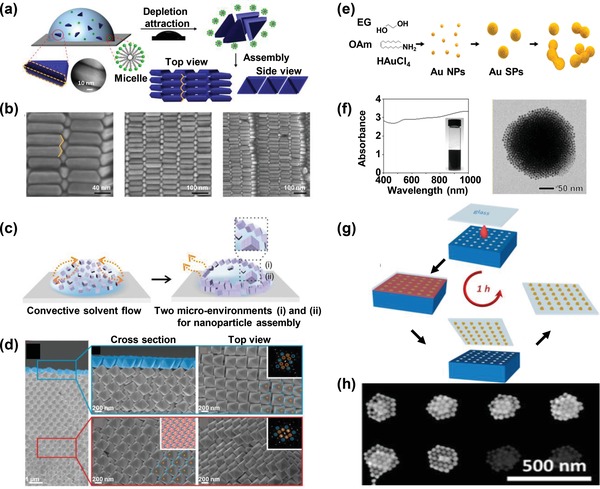
a) Beveled gold triangular prism assembly via droplet evaporation. b) SEM images of the i‐honeycomb lattice. Reproduced with permission.^52^ Copyright 2017, American Chemical Society. c) Formation of supercrystals with two unique structures. d) Cross‐sectional SEM images of the supercrystal assembled using octahedra as building blocks. Reproduced with permission.^29^ Copyright 2018, Nature Publishing Group. e) Formation of colloidal gold superparticles through self‐assembly of in situ–formed gold nanoparticles. f) Photograph, UV–vis spectrum, and TEM image of colloidal gold superparticles. Reproduced with permission.^53^ Copyright 2018, American Chemical Society. g) Schematic representation of the template‐assisted assembly of gold nanospheres into periodic arrays of well‐defined gold nanosphere clusters over large areas. h) SEM image of representative gold nanosphere clusters. Reproduced with permission.^54^ Copyright 2018, American Chemical Society.

Kim et al. used shape‐anisotropic nanoparticles (gold triangular prisms) as building blocks to form supercrystals on silicon substrates via a droplet evaporation method in presence of depletants (**Figure** **5**a,b).^52^ The resulting interlocking honeycomb brought all plasmonic surfaces into close proximity, and led to a strong SERS enhancement that was able to detect 10^−8^ m of rhodamine 6G. Dual‐structure supercrystals using polyhedral nanoparticles have recently been created via two self‐assembly microenvironments (Figure 5c,d).^29^ These dual‐structural supercrystals showed a 3.3‐fold higher SERS enhancement efficiency as compared to uniformly structured supercrystals. While considerable progress has been achieved in supercrystal synthesis, these methods do still require multiple time‐consuming steps (12 h to 1 week).

To speed up the synthesis of supercrystals, a simple one‐step synthesis method is highly desired. Kwon et al. developed a direct chemical synthesis of plasmonic black colloidal gold supercrystals with broadband absorption in visible and NIR regions (Figure 5e,f).^53^ The chemical synthesis involves self‐assembly of in situ–formed gold nanoparticles driven by solvophobic interactions between nanoparticles and solvent. Furthermore, Matricardi et al. proposed a scalable approach to fabricate plasmonic supercrystal arrays using the template‐assisted assembly of gold nanospheres with topographically patterned polydimethylsiloxane molds (Figure 5g,h).^54^ The whole fabrication process only requires 1 h. The supercrystals possess well‐defined collective plasmon modes that can be tailored from visible through NIR regions by simple modification of the lattice parameter via specific polydimethylsiloxane patterns. This process thus paves the way toward the synthesis of high‐yield, enhancement‐efficient, and reproducible supercrystals.

### 2.5 Nanostructures‐on‐Film

In the previous subsections, the geometry of plasmonic architectures is limited and strongly governed by the synthesis method, which in turn restricts further improvement of SERS performance. In contrast, nanostructures‐on‐film, defined as nanostructures formed on the surface of 2D substrates, could be artificially fabricated into flexibly shaped structures to achieve new desirable optical and electronic properties. Generally, nanostructures‐on‐film can be constructed using self‐assembly methods and lithography/template technologies.

#### 2.5.1 Self‐Assembled Nanostructures

**Figure 6:**
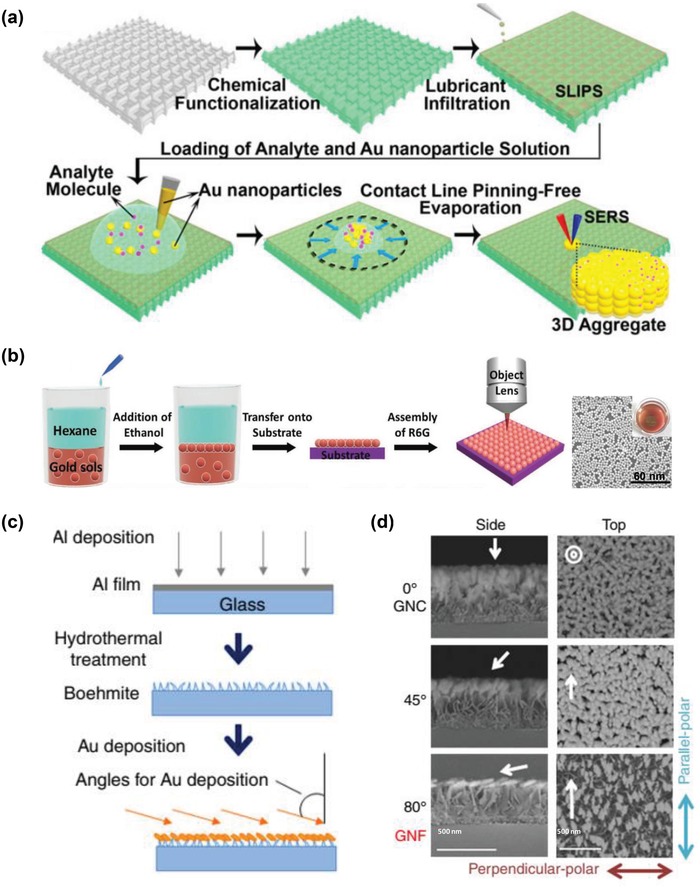
a) Schematic illustration of complete concentrations of analytes and gold nanoparticles within an evaporating liquid droplet via a slippery omniphobic substrate. Reproduced with permission.[qv: 55b] Copyright 2016, National Academy of Sciences (USA). b) Synthesis scheme, SEM image, and optical image (inset) of gold nanoparticle monolayers obtained from water/hexane interface for SERS measurements. Reproduced with permission.^56^ Copyright 2016, Wiley‐VCH. c) Nanofabrication of gold‐nanofève substrate (GNF). d) SEM images showing alterations in the quasiparallel alignment of anisotropic gold nanostructures at deposition angle at 0° (gold–nanocoral substrate, GNC), 45°, and 80° (GNF). Reproduced with permission.^58^ Copyright 2018, Nature Publishing Group.

Self‐assembly methods are simple and have the capability of generating a high yield of sub 10 nm gaps on 2D substrates for single‐molecule detection.^55^ For example, Yang et al. achieved a 3D hotspot matrix by evaporating a droplet of plasmonic nanoparticles on a hydrophobic substrate (**Figure** **6**a). The self‐assembled gold nanoparticle aggregates could sensitively detect trace amount (i.e., 10 × 10^−18^ m) of biological targets, including thymine, adenine, and bovine serum albumin.[qv: 55b] However, this platform might suffer from relatively poor signal reproducibility due to the random distribution of hot spots. To generate SERS substrates with a uniform distribution of hot spots, Si et al. proposed oil–water interfacial self‐assembly of gold nanoparticles on stretchable poly(dimethylsiloxane) substrates (Figure 6b),^56^ which created sub 1 nm gaps of higher density and reproducibility as compared to by using evaporation‐based synthesis methods. However, the homogeneous distribution of nanoparticles on the substrate is yet to be achieved, as evident from large void areas observed in scanning electron microscopy (SEM) images.

For detecting native biomolecules at the level of tumor cells and tissues, it is important to construct a large‐area SERS‐active substrate. Yamazoe et al. proposed a simple, reproducible, and cost‐effective strategy that used a self‐assembled nanostructure (i.e., boehmite) as a template for gold deposition.^57^ The gold nanoparticles were grown on random nanoarrays directly, and the resulting SERS substrate exhibited less than 11.2% signal intensity variation in detecting rhodamine 6G molecules across 121 spots over an area of 10 × 10 mm^2^. More importantly, this SERS substrate was capable of performing tissue imaging, over a maximum area of 24 × 24 mm^2^ and was sensitive to detect metabolic derangement of 9‐substituted adenine derivatives at an acute phase of brain ischemia.

Shiota et al. further improved the detection sensitivity through better control of nanostructure geometries by deposing gold nanoparticles from an oblique direction at an angle of 80° to the self‐assembled boehmite substrates, leading to a highly porous and anisotropic island growth (Figure 6c,d).^58^ With this gold‐nanofève SERS substrate, they successfully identified tumor boundaries in liver tissues based on the distinct molecular vibration fingerprints of tissue metabolites. Following this study, the automated pathological diagnosis of cancer is currently underway through further integration with digital image processing algorithms and prelearning by an experienced pathologist.

#### 2.5.2 Ordered Nanostructures

Lithography and template methods are routinely used in the fabrication of highly ordered plasmonic nanostructure arrays. For example, electron‐beam lithography has advantages in fabricating a wide range of 2D periodic geometries with ultrafine features, good reproducibility, and large‐scale uniformity, such as the nanoantenna dimer arrays,^59^ ordered nanopillars,^60^ nanostar dimer arrays,^61^ and 3D multipetal flower arrays.^62^ However, electron‐beam lithography is inapplicable for the mass production of nanostructure‐on‐film due to low efficiency in time and cost. Recently, the construction of nanostructure arrays with uniform nanogaps (10–20 nm) has been engineered by many alternative time‐ and cost‐effective technologies, including template‐assisted assembly,^54^ laser interference lithography,^63^ and laser‐assisted nanoreplication methods.[qv: 60a]

**Figure 7:**
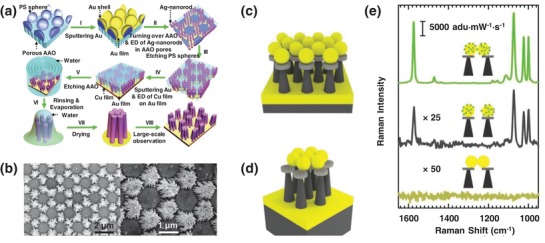
a) Schematic of fabrication procedure and b) SEM images of ordered arrays of silver nanorod bundles. Reproduced with permission.[qv: 64c] Copyright 2016, Wiley‐VCH. Nanohoodoos topped by c) single gold nanoparticles and d) gold nanoparticle clusters formed during evaporation of solvent. e) SERS spectra of thiophenol obtained from these SERS substrates. Reproduced with permission.^65^ Copyright 2018, Wiley‐VCH.

With the advancement of facile fabrication techniques, various “state‐of‐the‐art” nanostructures have been designed to achieve high‐efficiency SERS measurements. One type of newly designed nanostructures is leaning nanopillar arrays that cluster together to form narrow interparticle gaps (1–10 nm) on the top of nanopillars.^64^ These structures were created through the facile solvent wetting (Wenzel state) method, which provides attractive capillary forces among nanopillars (**Figure** **7**a,b).[qv: 64c] A similar nanostructure type has also been synthesized by gold nanoparticles sliding on nanohoodoos and forming clusters via deposition and evaporation of deionized water (Figure 7c–e).^65^ Although these close‐packed nanoparticles or nanopillars (usually at sub 10 nm) dramatically enhance SERS signaling, they are often subjected to poor reproducibility due to uncontrollable aggregation. In addition, the vertical spreading of solution droplets often results in random adsorption of molecules across the entire nanostructure surface which has varying levels of SERS activities, thus eventually leading to poor experimental reproducibility and detection sensitivity.

**Figure 8:**
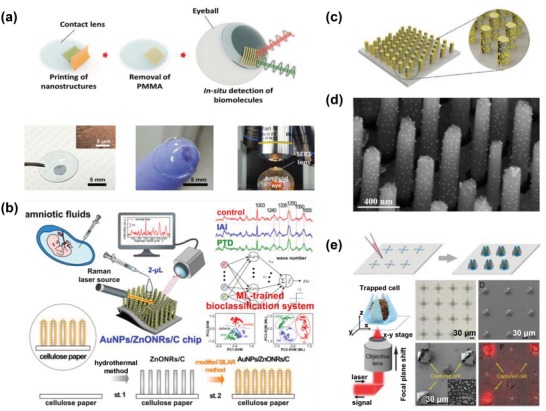
a) Fabrication of SERS contact lens and demonstration of glucose detection. Reproduced with permission.^66^ Copyright 2016, Wiley‐VCH. b) Gold nanoparticle–decorated ZnO nanorods grown on cellulose paper for SERS analyses of amniotic fluids to detect prenatal diseases. Reproduced with permission.^67^ Copyright 2018, American Chemical Society. c) Illustration and d) SEM image of gold nanoparticle–conjugated nanorod arrays. Reproduced with permission.^68^ Copyright 2017, American Chemical Society. e) Gold nanostar–functionalized mechanical trap for 3D surface molecular profiling of single live cells. Reproduced with permission.^69^ Copyright 2017, Wiley‐VCH.

To generate a nanostructure design with homogeneous distributions of hot spots in 3D space, Jeong et al. developed 3D cross‐point plasmonic nanoarchitectures containing dense hot spots via an optimized solvent‐assisted nanotransfer printing technology (**Figure** **8**a).^66^ The resulting nanoarchitectures achieved an EF of 4.1 × 10^7^ through integration of an in‐plane coupling effect in the sub 20 nm nanogaps between sequentially transfer‐printed nanowires, and an out‐of‐coupling effect at the cross‐points where two nanowires were closely stacked. More interestingly, this substrate was further engineered as soft SERS contact lens mounted on an artificial glass eye for in situ detection of 0.1 × 10^−3^ m glucose in solution (which corresponds to the glucose level in tears of diabetes patients).

An alternative nanostructure design with 3D distributions of hot spots is gold‐nanoparticle‐conjugated hierarchic nanostructures. Such hierarchic nanostructures could be tailored to enable measurements of either nanometer‐sized (e.g., circulating tumor NAs) or micrometer‐sized (e.g., CTCs) biomolecules. For the measurement of small biomolecules, Kim et al. developed a paper‐based SERS sensor chip—gold nanoparticles were decorated on zinc oxide (ZnO) nanorod arrays that were vertically grown on cellulose paper (Figure 8b).^67^ A hydrothermal method was utilized to align the dense vertical growth of ZnO nanorod arrays on porous cellulose paper, and an optimized successive ionic layer adsorption and reaction method was employed for the decoration of gold nanoparticles on ZnO nanorods. The EF of this SERS substrate was calculated to be ≈1.25 × 10^7^, with an excellent reproducibility of <6% variability. The authors applied this chip for the detection of trace amounts of human amniotic fluids and identified different types of prenatal diseases with >92% clinical sensitivity and specificity.

For the analysis of micrometer‐sized biotargets, Lin et al. fabricated a gap‐independent gold nanoparticle–functionalized Si nanorod array for ultrasensitive SERS detection of amyloid aggregates. This gold nanoparticle–functionalized Si nanorod array produced a long decay length of >130 nm (Figure 8c,d).^68^ For microlevel analysis, Jin et al. also reported a mechanical trap SERS platform for simultaneous capture and 3D microscopic mapping of cell surfaces (Figure 8e).^69^ The functionalization of inner surfaces of glasslike mechanical traps with gold nanostars and the conformal contact of cell membranes enable sensitive, nonperturbative, and multiplex 3D surface molecular analysis of single cells (not achievable by use of a 2D SERS substrate). Additionally, the mechanical trap offers a stable orientation of single cells, which allows a 3D microscopic analysis of intrinsic molecular signatures (e.g., lipids and proteins) of cell membranes to be performed in diverse environments.

**Figure 9:**
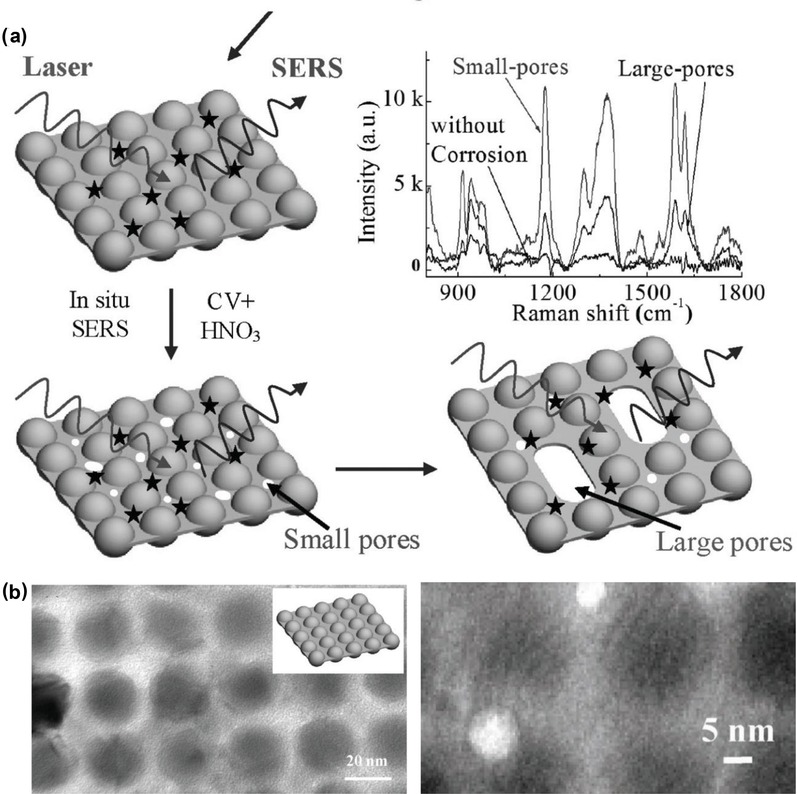
a) Illustration of a “nanopore‐in‐nanogap” hybrid structure for SERS measurements. b) TEM images of 2D silver nanoparticle supercrystals (left) and “nanopore‐in‐nanogap” hybrid arrays (right). Reproduced with permission.^70^ Copyright 2017, Wiley‐VCH.

In addition to nanostructures which possess a single type of hot spot enhancement mode (e.g., nanorod arrays), hybrid nanostructures (that comprise two or more different nanostructures) are getting increasing attention due to the innovative integration of multiple plasmonic modes, or integrated plasmonic and optical cavities, for enhanced electromagnetic coupling. For example, Ma et al. developed a plasmonic silver “nanopore‐in‐nanogap” hybrid structure (**Figure** **9**).^70^ They first synthesized large‐area 2D silver nanoparticle supercrystals with uniform nanogaps of ≈3 nm using 3D ordered mesoporous silica as a hard template together with a nanocasting process. An etching process was then performed to create ultrasmall nanopores of sub 10 nm in diameter on 2D silver nanoparticle supercrystals. The resulting “nanopore‐in‐nanogap” hybrid nanostructures thus possessed a combined nanopore and nanogap plasmon modes, which provided an additional electromagnetic enhancement for the ultrasensitive detection of 4‐aminothiophenol down to 0.1 × 10^−15^ m. Currently, there are many other hybrid nanostructures in reports, including a split‐wedge antenna with 1 nm gaps,^71^ vertically coupled complementary antenna,^72^ bimetallic 3D nanostar dimers in ring cavities,^73^ and a SERS substrate with a 3D arrangement of nanohole array and an optical cavity.^74^

### 2.6 Microchanneled Nanostructures

**Figure 10:**
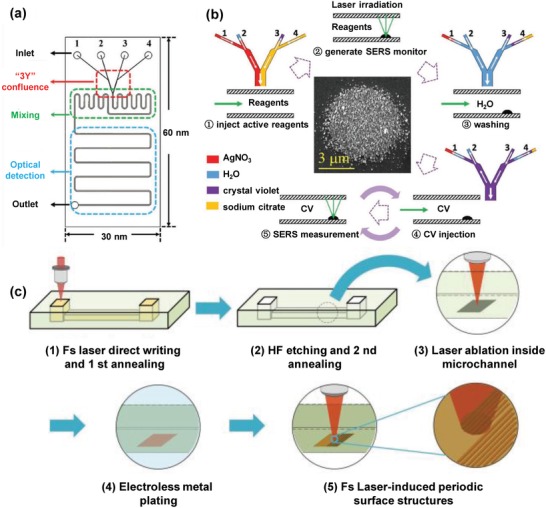
a) Illustration of a microfluidic chip with three modules including an inlet, mixing, and SERS detection. b) Procedure of photoinduced growth of silver nanoaggregates and in situ SERS measurements. Reproduced with permission.[qv: 24e] Copyright 2017, Wiley‐VCH. c) Procedure used to fabricate a 3D microfluidic SERS chip by all‐femtosecond‐laser processing. Reproduced with permission.[qv: 75d] Copyright 2018, Wiley‐VCH.

Nanostructures fabricated directly within a microchannel (termed as “microchanneled nanostructures”) have recently been developed for the microfluidic SERS detection. This fabrication method aims to replace the need of having to inject colloidal plasmonic nanoparticles into microfluidic channels, which demands elaborate microfluidic design and careful control of the flow rate. Currently, there are limited research reports on the fabrication of plasmonic nanostructure arrays in closed microfluidic channels.[qv: 24e,75] These studies could be classified into two groups according to nanostructure fabrication methods: i) synthetic chemistry and ii) nanolithography. Yan et al. proposed a synthetic chemistry‐based strategy that utilized two‐step photoreduction to synthesize silver nanoparticle aggregates directly in a microfluidic channel, followed by in situ single‐molecule SERS measurements (**Figure** **10**a,b).[qv: 24e] The resulting SERS substrates enabled effective detection of different dye molecules (e.g., rhodamine 6G) at a low concentration of 10^−13^ m, and single‐molecule SERS measurements for up to ≈50% of all molecules. Bai et al. applied the all‐femtosecond‐laser‐processing technique to engineer a 2D periodic plasmonic nanostructures within 3D glass microfluidic channels (Figure 10c).[qv: 75d] The geometry of deposited plasmonic nanostructures could be controlled through adjustments of laser ablation parameters, laser scanning schemes, and metallization time. Engineered nanostructures that consist of layered copper–silver nanodots could be fabricated at a size as small as 25% of the laser wavelength, thus allowing for the detection of ≈10^−8^ m of rhodamine 6G with a relative standard deviation of 8.88%.

### 2.7 Comparisons of Different Plasmonic Nanomaterials

**Figure 11:**
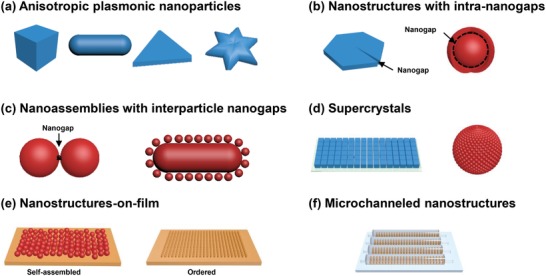
a–f) Schematical illustration of different types of plasmonic nanostructures.

For better comparison, we have schematically overviewed (**Figure** **11**) the representative structures of each type of plasmonic nanomaterials. Their advantages and disadvantages have been compared and summarized in **Table** **1**. We found that most plasmonic nanomaterials only tested their performance using common Raman reporters, which may not provide sufficient justification for their suitability for biomolecule detection. As such, to facilitate the applications of these plasmonic nanomaterials in SERS‐based liquid biopsy sensing, it is encouraged to include different types of biomolecules (e.g., protein and NA) for evaluation of nanomaterial detection performance.

**Table 1:** Representative SERS plasmonic nanomaterials as well as their synthesis methods, key features (i.e., enhancement factor (EF), gap size, signal reproducibility (SR)), detection targets, and potential applications (Cyt C: Cytochrome c; 4‐MPY: 4‐Mercaptopyridine; R6G: Rhodamine 6G; 2,4‐D: 2,4‐Dichlorophenoxyacetic acid; CV: Crystal violet; 4‐ATP: 4‐Aminothiophenol)

Plasmonic nanomaterials	Synthesis	Key features	Targets	Potential applications
Anisotropic plasmonic nanoparticles				
Silver nanocubes^21^	PVP‐mediated synthesis	EF ≈ 2 × 10^5^ SR < 10%	Cyt C (5–10 × 10^−9^ m)	SERS substrates for direct SERS detection SERS nanotags for barcoding
Nanodot‐decorated gold–silver alloy nanoboxes^38^, ^39^	Template‐free method	EF ≈ 10^6^	sPD‐1 (6.17 pg mL^−1^), sPDL‐1 (0.68 pg mL^−1^), sEGFR (69.86 pg mL^−1^)	
Nanostructures with intra‐nanogaps				
Gold–silver alloy nanoislands^42^	Partial surface passivation	EF ≈ 5 × 10^6^	CV	SERS substrates for direct SERS measurements of nanosized biomolecules (accessible intra‐nanogaps) Quantitative SERS analyses (enclosed intra‐nanogaps)
Silver nanoplates with intra‐nanogaps^43^	Seed‐mediated growth	Gap: 90 nm in length, 2 nm in width	2‐naphthalenethiol (1 × 10^−9^ m)	
Core–shell nanospheres with enclosed nanogaps[qv: 23d]	Polydopamine‐enabled approach	EF = 8.8 × 10^7^ Gap = 2 nm	Bacteria (10^2^ CFU mL^−1^)	
Nanoassemblies with interparticle nanogaps				
Gold nanosphere dimers^48^	Thiolated linker‐free synthesis	EF = 5 × 10^5^ Gap = 0.76 nm	4‐MPY	Sensor platform for nanosized biomolecules SERS nanotags for biomolecule labeling
Jenus gold–organosilica dimers^49^	Self‐assembly	EF = 6.6 × 10^6^ Gap = 0.7–0.9 nm	R6G (10 × 10^−9^ m)	
Gold nanostar dimers[qv: 24b]	DNA origami– based method	EF ≈ 10^9^ Gaps = 7 or 13 nm	Texas red dye	
Gold pyramids[qv: 24g]	DNA frame– based method	EF ≈ 10^5^ Multigaps	Telomerase (6.2 × 10^−15^ IU)	
Supercrystals				
Gold prism assemblies^52^	Droplet evaporation	EF = 5.5 × 10^4^ Gap = 5–6 nm	R6G (10 × 10^−9^ m)	Plasmonic substrates for direct SERS measurements
Colloidal gold supercrystals^53^	Direct chemical synthesis	EF = 17.4 Gap = 2 nm	Graphene	
Nanostructures‐on‐f ilm				
Gold‐nanofève substrates^58^	Deposition	EF ≈ 10^7^ SR ≈ 11.0%	Metabolites in human tissue	Qualitative analysis of microsized or bigger subjects via direct SERS measurements
Gold nanopillar bundle arrays[qv: 64c]	Solvent wetting method	EF = 10^8^ Gap = 2 nm SR < 10%	Methyl parathion (21.5 × 10^−9^ m), 2,4‐D (61.9 × 10^−9^ m)	Ultrasensitive, quantitative analysis of biomolecules via direct SERS measurements
3D cross‐point plasmonic nanoarchitectures^66^	Nanotransfer printing	EF = 4.1 × 10^7^ Gap < 20 nm SR < 10%	Glucose (0.1 × 10^−3^ m)	
Gold nanosphere‐conjugated hierarchic nanostructures^67^	Bottom‐up technique	EF = 1.25 × 10^7^ Gap = 1–2 nm SR < 6%	CV (100 × 10^−12^ m), Human amniotic fluids	
Gold nanosphere–functionalized Si nanorod arrays^68^	Top‐down technique	EF ≈ 10^7^ Gap > 130 nm SR 3.9–7.2%	R6G (0.1 m), amyloid‐β fibrils	
Nanopore‐in‐nanogap silver nanostructures^70^	Nanocasting	Gap: ≈3 nm SR < 12%	CV (10 × 10^−15^ m), 4‐ATP (0.1 × 10^−15^ m)	
Microchanneled nanostructures				
2D periodic metal nanostructures[qv: 75d]	Femtosecond laser–assisted etching	EF = 7.3 × 10^8^ Gap = 50 nm SR = 8.88%	Cd^2+^ ions (10 ppb)	Real‐time SERS measurements

## 3 Plasmonic Nanomaterial‐Based SERS Liquid Biopsy Analyses

**Figure 12:**
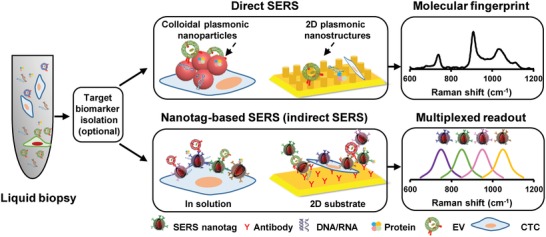
General detection principles for various types of liquid biopsy biomarkers using plasmonic nanomaterial‐based SERS technologies.

SERS analysis based on innovatively engineered plasmonic nanomaterials (as reviewed in the preceding section) can potentially benefit clinical liquid biopsy analyses with exemplary detection sensitivity and multiplexity attributes.^76^ Generally, SERS‐based liquid biopsy biosensing strategies could be classified into two classes (**Figure** **12**): i) direct SERS and ii) nanotag‐based SERS (indirect SERS). Direct SERS detects molecular fingerprint information of biomarkers and could be performed by directly incubating target samples with plasmonic nanomaterials. Indirect SERS normally relies on the SERS nanotags for barcoding target biomarkers, which is feasible for highly multiplexed detection. In this section, we first summarize the clinical value, traditional detection methods, and current analysis challenges of various liquid biopsy biomarkers (i.e., CTCs, tumor‐derived EVs, circulating cancer proteins, and circulating tumor NAs). We then focus on how plasmonic nanomaterial‐based SERS technologies have been explored to overcome current analysis challenges, as well as insights to promote clinical translation for various liquid biopsy biomarkers.

### 3.1 Circulating Tumor Cells

CTCs are tumor cells that have been passively shed into the bloodstream from a primary tumor and/or metastatic lesions. Based on the “seed and soil” theory, a small portion of CTCs (<0.01%) may seed a secondary tumor (metastasis).^77^ Clinical evidence has demonstrated the presence of CTCs in various metastatic carcinomas (e.g., melanoma, breast, and prostate).^78^ It has also been reported that CTCs can be used for studying protein localization and cell morphology of tumor cells, which cannot be provided by other liquid biopsy biomarkers.^2^, ^120^ Additionally, many studies have indicated that the abundance of CTCs is associated with the tumor burden and could serve as an indicator of treatment responses.^79^ However, there are only as few as 1–10 CTCs mixed with ≈7 × 10^6^ leukocytes and ≈5 × 10^9^ erythrocytes in 1 mL of blood.^80^ Furthermore, CTCs are extremely heterogeneous,^81^ thus posing further technical challenges for CTC detection and characterization. As such, it is fundamentally important to develop technologies that can identify CTCs sensitively, specifically, and reproducibly.

Considering the rarity of CTCs against the background of blood cells and other biomolecules in the blood of a cancer patient, CTC isolation methods are often performed prior to downstream analyses, either by i) positive CTC isolation which selectively enriches CTCs from the blood or ii) negative CTC isolation based on the depletion of blood cells.

Generally, positive CTC isolation methods can be classified based on whether they exploit the biological or physical properties of CTCs, alternatively referred to as ligand‐dependent or ligand‐independent approaches, respectively. The most common ligand‐dependent method is using magnetic particles conjugated with cancer‐specific antibodies. The CellSearch system (Veridex, LLC) is the only Food and Drug Administration (FDA)‐approved CTC detection technology for clinical use. This system magnetically isolates CTCs expressing epithelial cell adhesion molecule (EpCAM) from whole blood, followed by CTC identification via immunofluorescence imaging for positive intracellular cytokeratin and negative CD45 expression. The LOD achieved by the CellSearch system is 1 cell per 7.5 mL blood. However, the CellSearch system is prone to overlooking CTCs that have low EpCAM expression due to the epithelial–mesenchymal transition (EMT) process and/or nonepithelial origins.^82^ Additionally, as magnetic particles used for CTC isolation are mainly micrometer‐sized magnetic beads,^46^ they have a relatively low surface‐to‐volume ratio to provide an efficient binding affinity toward CTCs and tend to precipitate out of blood during the sample incubation process. In contrast, ligand‐independent CTC enrichment approaches are based on physical properties of CTCs, such as the size‐based ISET (Isolation by SizE of Tumor/Trophoblastic Cells). The LOD achieved by ISET is 1 cell mL^−1^ blood.^83^ However, there are caveats which limit the efficiency of size‐exclusion enrichment approaches, such as the variable stiffness and size of EMT‐related CTCs.

Alternatively, negative CTC isolation methods based on blood cell depletion have been proposed to isolate a collection of diverse CTCs by reducing the loss of CTCs that express inadequate ligand‐specific biomarkers, and/or are small‐sized (i.e., critical limitations present in the positive isolation of specific CTC subpopulations). Negative CTC isolation normally includes two steps: i) depletion of erythrocytes via erythrocyte lysis or density gradient centrifugation and then ii) depletion of CD45^+^ leukocytes using the MACS MicroBead technology (Miltenyi Biotec, Germany) or EasySep cell isolation kit (STEMCELL Technologies Inc., Canada).

Following CTC isolation, the captured CTCs could then be analyzed via protein‐based techniques such as fluorescence‐assisted cell sorting or NA‐based techniques such as PCR.^84^ Fluorescence‐assisted cell sorting provides high‐throughput analyses of cell phenotypes but typically requires a large number of cells (1 target cell in 1000 nontarget cells)^85^ and four lasers for 17‐plex detection.^86^ Mass cytometer (CyTOF) overcomes these limitations;^87^ however, it is incapable of collecting live cells for downstream analyses. While PCR can quantify the relative expression of target transcripts within low quantities of CTCs, it is difficult to provide accurate CTC count and morphological information. Thus, an innovative method is highly desired for sensitive CTC detection and multiplex phenotypic characterization.

#### 3.1.1 CTC Enumeration

Integration of CTC isolation strategies with SERS readouts provides a sensitive platform for CTC detection. Ruan et al. have demonstrated that a combination of magnetic bead–based positive isolation of CTCs and triangular silver nanoprism‐based SERS detection yielded better sensitivity and specificity than the SERS detection alone, and improved the LOD from 5 cells mL^−1^ to 1 cell mL^−1^ from blood samples.^88^

SERS nanotags of high performance are essential for achieving ultrasensitive CTC detection. Wu et al. recently estimated the CTC detection sensitivity achieved by using gold nanospheres, gold nanorods, and gold nanostars of different shapes but similar particle size and modifications.^89^ They concluded that gold nanostars (conjugated with 4‐mercaptobenzoic acid Raman reporters and cancer‐cell‐specific ligand folic acids, and stabilized with reductive bovine serum albumin) showed a higher analytical sensitivity than spherical gold nanoparticles and gold nanorods, with an LOD of 1 cell mL^−1^ in the peripheral rabbit blood.

**Figure 13:**
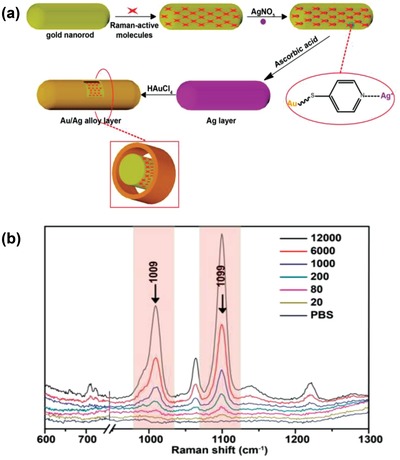
a) Construction of core–shell plasmonic nanorods with encapsulation of Raman reporters. b) SERS spectra of a different number of MCF7 cells from 20 to 12 000 spiked in blood mimicking fluid. Reproduced with permission.^91^ Copyright 2017, American Chemical Society.

To avoid external environment‐associated effects such as unintended replacement of Raman reporters and particle aggregation,^90^ core–shell plasmonic nanoparticles have been applied for accurate CTC enumeration. Zhang et al. exploited core–shell plasmonic nanorods with the encapsulation of Raman reporters to detect as low as 20 MCF7 cells directly in simulated blood (**Figure** **13**).^91^ Additionally, the morphologies of core–shell plasmonic nanorods could be tuned to tailor LSPR into the NIR region, which further amplified SERS signals for sensitive CTC enumeration.

#### 3.1.2 CTC Phenotyping

**Figure 14:**
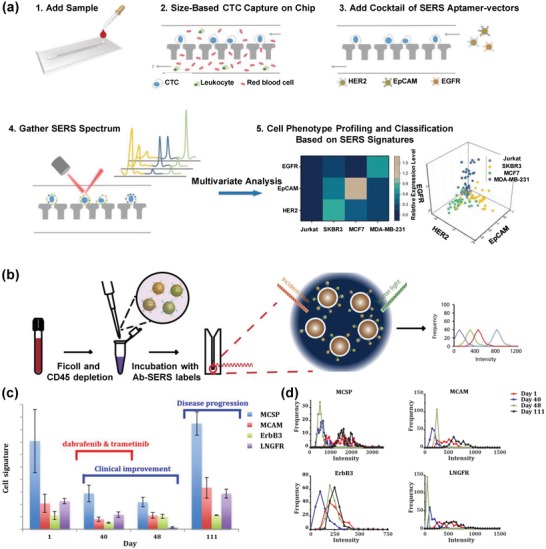
a) Workflow of a size‐based microfluidic SERS platform for CTC capture, cell phenotype profiling, and SERS signature‐based classification. Reproduced with permission.^92^ Copyright 2018, Wiley‐VCH. b) Schematics of experimental workflow for CTC detection and characterization with a multiplex SERS nanotag system. c) CTC phenotypic evolution of patient 1 according to days of treatment. d) Diversity of CTC surface marker expression in response to treatment. Reproduced with permission.^93^ Copyright 2018, Nature Publishing Group.

The advances in isolation strategies and plasmonic nanomaterials have also led to new insights into the biology of CTCs, such as CTC heterogeneity. Zhang et al. developed a size‐based microfluidic SERS platform for in situ profiling of cell membrane proteins and identification of cancer subpopulations (**Figure** **14**a).^92^ The 12 µm microfluidic filters efficiently sieved CTCs from blood based on the size discrepancy between CTCs and blood cells, resulting in capture rates of 87 ± 8%, 93 ± 6%, and 87 ± 8% for breast cancer cells SKBR3, MCF7, and MDA‐MB‐231, respectively. The combination of the multiplex SERS nanotag labeling system and categorization algorithm‐partial least square discriminant analysis enabled the accurate classification of cancer subpopulations.

Tsao et al. recently developed a SERS‐based CTC characterization strategy that utilized a multiplex SERS nanotag system for simultaneous phenotyping of multiple CTC surface biomarkers, and further applied it for monitoring the phenotypic evolution of CTCs during melanoma treatment (Figure 14b–d).^93^ This strategy offered a sensitive CTC detection of ≈10 cells from 10 mL of blood. More importantly, comprehensive CTC heterogeneity characterization could be simply achieved via the analysis of SERS signal variations caused by CTCs undergoing Brownian motion in solution (Figure 14b). With this strategy, they observed cell heterogeneity and phenotypic changes of three melanoma cell lines during molecularly targeted therapy. Furthermore, the analysis of CTC phenotypic evolution of ten stage‐IV melanoma patients receiving immunological or molecular targeted therapies indicated drug‐resistant CTC clones possessing different potential clinically actionable phenotypes. To achieve a further in‐depth and accurate analysis of CTCs, a higher‐multiplex SERS nanotag labeling system is desired. It is believed that the integration of numerous biomarkers could further improve the understanding of CTC biological properties, and thus has the potential to improve treatment management.

#### 3.1.3 Outlooks for SERS‐Based CTC Analysis

In conclusion, the development of advanced plasmonic nanomaterials and CTC isolation strategies has enabled ultrasensitive CTC enumeration (LOD: 1 cell mL^−1^ blood) for cancer diagnosis.^88^, ^92^, ^93^, ^94^ A recent SERS platform which demonstrated a simultaneous 4‐plex signal readout using one excitation laser has further enabled the investigation of CTC heterogeneity,^93^ which is a limitation of commercial CTC detection assays/systems (e.g., CellSearch system, ISET, and fluorescence‐assisted cell sorting). As such, the development of SERS‐based CTC technologies (**Table** **2**) might facilitate the clinical applications of CTC biomarkers by providing a sensitive, comprehensive CTC characterization tool. However, most SERS‐based CTC technologies are limited to the detection of cell‐surface biomarkers; therefore, developing strategies for CTC intracellular biomarker detection (some technologies have been applied for the cell line model^95^) might provide new insights into their characteristics. Moreover, current SERS‐based CTC detection strategies mainly focused on improving the assay sensitivity and specificity, while the comprehensive downstream analysis of CTC biology has largely been ignored. As not all CTCs possess the metastatic potential for detection, simple enumeration of CTCs without molecular characterization may lead to inaccurate clinical diagnosis and consequences. As such, the next frontier in the SERS‐based CTC field may progress toward comprehensive CTC characterization using constantly evolving plasmonic nanomaterials, which could help elucidate the clinical value of CTCs as molecular biomarkers and therapeutic targets. To promote ultimate clinical applications of CTCs as a predictive and prognostic biomarker, we also suggest that demonstrations of plasmonic nanomaterials for SERS‐based CTC analyses should include i) more patient sample testing to fully evaluate usage feasibility in highly variable clinical samples from different patients, and ii) further evaluation of relevance between CTC analysis data and clinical outcomes to investigate assay clinical performance.

**Table 2:** Comparison of different SERS strategies for CTC analysis

CTC Assays	Sensitivity	Limitations	Purposes	Applications
Isolation‐free methods integrated with single‐plex readouts^139^	5 cells mL^−1^ blood	Lower detection sensitivity	Enumeration	Cancer diagnosis
Ligand‐dependent positive isolation integrated with single‐plex readouts^88^, ^94^	1 cell mL^−1^ blood	Ligand dependent	Enumeration	Cancer diagnosis
Size‐dependent positive isolation integrated with 3‐plex readouts^92^	Not provided	Prone to losing small‐sized CTCs and retaining large‐sized leukocytes	Phenotyping	Cancer diagnosis
Negative isolation integrated with 4‐plex readouts^93^	1 cell mL^−1^ blood	Require two depletion steps including erythrocyte and CD45^+^ leukocyte depletion	Heterogeneity evaluation	Treatment monitoring

### 3.2 Tumor‐Derived Extracellular Vesicles

EVs are lipid‐bilayer‐enclosed membrane vesicles of 30–10 000 nm in diameter and secreted by essentially all cell types (e.g., blood cells, immunocytes, and tumor cells). EVs can be classified into two major subclasses: exosomes (30–100 nm in diameter) which are formed by the inward budding of the endosomal membrane during maturation of multivesicular endosome, and microvesicles (50–10 000 nm in diameter) that are shed from the plasma membrane surface via outward budding.^96^

EVs are of interest for diagnostic and prognostic applications as they contain various molecules (e.g., NAs and proteins) derived directly from secreted cells. EVs can also be used to track inflammatory responses, as well as stromal and other systemic changes (which are not derivable from CTCs or circulating tumor NAs).^2^ Additionally, EVs can be easily sampled as they are present in various body fluids (e.g., blood and urine).^97^, ^120^ However, sensitive and specific detection of tumor‐derived EVs is difficult due to the i) abundant background presence of EVs secreted from nonneoplastic cells as well as other nonvesicular materials,[qv: 1b,98] and ii) inherent EV heterogeneity in biophysical characteristic and composition.^97^ Furthermore, the current lack of a standardized EV isolation and analysis workflow makes the clinical translation of EVs even challenging.

To date, main EV isolation methods include ultracentrifugation (e.g., differential ultracentrifugation and density gradient ultracentrifugation), size exclusion (e.g., ultrafiltration and size exclusion chromatography), polymer‐facilitated precipitation (e.g., ExoQuick, Total Exosome Isolation Kit), and immunoaffinity isolation.^97^, ^99^ Differential ultracentrifugation remains the most commonly used technique for EV isolation but easily causes vesicle aggregation and co‐isolates protein contaminants. Density gradient ultracentrifugation provides relatively pure EVs through overnight centrifugation on sucrose or iodixanol gradient; however, it is time‐consuming, low yielding, and operator dependent. Size exclusion methods are user‐friendly, straightforward, and gentler isolation alternatives to ultracentrifugation methods. The purity of resulting EVs is also similar to that of density gradient ultracentrifugation. Contrarily, polymer‐facilitated precipitation approaches can be performed via commercially available polymeric precipitation mixtures which are technically facile and time‐saving; however, the purity of EVs produced by polymer‐facilitated precipitation methods is generally poor. Lastly, immunoaffinity‐based methods allow the isolation of EVs bearing specific surface biomarkers, thus enabling the interrogation of EV subpopulations of interest.

Following EV isolation, downstream analyses of EVs include i) nanoparticle tracking analysis (NTA) and tunable resistive pulse sensing (TRPS) for measuring physical properties and concentrations; ii) western blot, ELISA, and liquid chromatography‐tandem–mass spectrometry for protein phenotyping; and iii) PCR and NGS for NA profiling. Nevertheless, these techniques normally require a large number of EV samples and are difficult for the in‐depth study of EV heterogeneity. As such, it is in great demand to develop a sensitive, multiplex technology that only requires a small number of EV samples for comprehensive EV analyses.

#### 3.2.1 EV Subpopulation Differentiation

**Figure 15:**
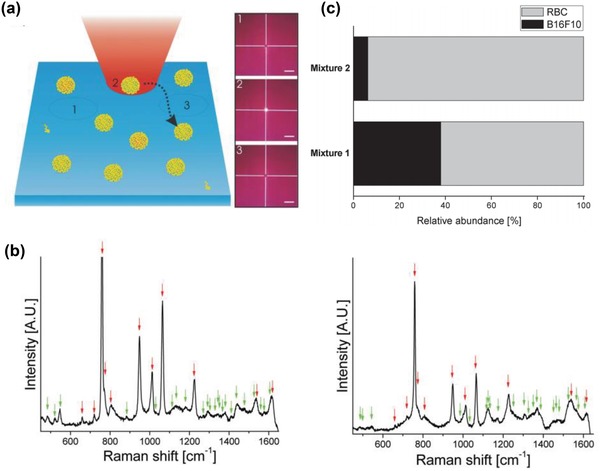
a) Schematic diagram of SERS measurements of gold nanoparticle–coated EVs. b) SERS spectra of EVs derived from B16F10 melanoma cells (left) and erythrocytes (right). Red arrows indicate peaks arising from the 4‐dimethylaminopyridine coating on the surface of gold nanoparticles, and green arrows are supposed to be peaks relevant to EVs. c) The composition of two EV mixtures determined by SERS measurements in combination with partial least squares discriminant analysis. Mixture 1 and Mixture 2 contain 51% and 21% of B16F10 melanoma‐derived EVs among erythrocyte‐derived EVs, respectively. Reproduced with permission.[qv: 100c] Copyright 2016, Wiley‐VCH.

Presently, the integration of EV isolation strategies and SERS technology has enabled differentiation of EVs derived from tumor and normal cells based on unique EV molecular components (e.g., cholesterol content, surface protein expression, and phospholipids).^100^ Stremersch et al. performed a direct SERS measurement to distinguish EVs of different cellular origins, in which gold nanoparticles were deposited on the surface of EVs that were isolated with density gradient ultracentrifugation (**Figure** **15**a).[qv: 100c] This strategy together with the partial least squares discriminant analysis successfully quantified the composition of EV mixtures derived from melanoma cells and erythrocytes (Figure 15b,c). Nevertheless, the feasibility of this strategy for cancer diagnosis still needs further clinical validation using real clinical samples.

Park et al. performed bulk SERS detection on EVs which were purified via ultrafiltration and size exclusion chromatography.^102^ The SERS substrates were fabricated by drying CuSO_4_‐induced aggregated gold nanoparticles over the cover glass. Further statistical analysis discriminated EVs derived from lung cancer cells and normal cells with a sensitivity of 95.3% and a specificity of 97.3%. The authors then applied this method to clinical samples obtained from two lung cancer patients and two healthy individuals. However, the differentiation of EVs derived from patient and normal blood samples is yet to be achieved, partially due to i) artificial signal variations caused by the random distribution of hot spots and EVs, and ii) rare tumor‐derived EVs being masked by the abundant background of EVs derived from normal cells.

To minimize aggregation‐induced signal variations and further improve SERS performance, plasmonic nanomaterials with well‐ordered nanostructures have been applied in direct SERS detection of EVs.[qv: 100a,b] Lee et al. developed a thin silver film–coated nanobowl SERS substrate for EV measurements in solution.[qv: 100b] The nanobowl‐structured substrate provided a uniform distribution of hot spots for reproducible EV measurements. Using this substrate, they monitored the development of new SERS characteristic peaks during the drying process, which enabled the analysis of exosomal membrane composition and contents within EVs. Ertsgaard et al. developed an integrated nanogap‐structured platform that combines subvolt dielectrophoresis trapping, gold nanoparticles, and SERS readouts for the detection of biological nanoparticles.[qv: 100a] This platform was capable of isolating suspended sub 100 nm EVs such that nanovesicles were trapped along the gap together with bare gold nanoparticles to boost local electromagnetic fields. However, the performance of this platform has only been evaluated in liposomes with the encapsulation of Raman reporter 4‐mercaptopyridine.

**Figure 16:**
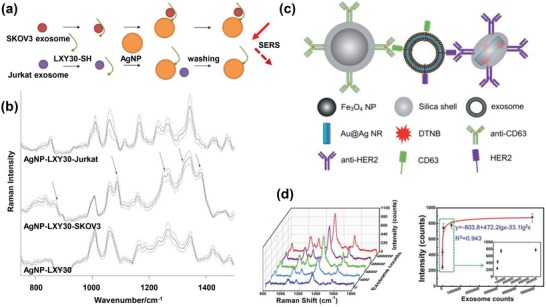
a) Schematic diagram of selective SERS detection of EVs derived from SKOV3 cells using silver nanoparticles conjugated with thiolated peptide ligands (LXY30‐SH) specific to α3ß1 integrin. b) Background SERS spectra and SERS spectra of LXY30‐SH conjugated silver nanoparticles for the detection of EVs from SKOV3 and Jurkat (control) cells. Reproduced with permission.[qv: 101a] Copyright 2017, Wiley‐VCH. c) Illustration of a SERS‐based EV detection method using magnetic nanoparticles as EV capture substrates, and gold core–silver shell nanorods as SERS nanotags. d) SERS spectra (left) and peak intensities at 1327 cm^−1^ (right) obtained with different amounts of EVs (from 4.88 × 10^3^ to 4.88 × 10^6^). Reproduced with permission.[qv: 101d] Copyright 2016, The Royal Society of Chemistry.

The identification and quantification of specific EV subpopulations have also been achieved by plasmonic nanomaterials conjugated with target‐specific ligands.^101^ Lee et al. performed a selective SERS analysis of EV subpopulations by using a thiolated peptide ligand that was bound to the surface of silver nanoparticles (**Figure** **16**a).[qv: 101a] The detectable EV‐specific SERS peaks indicated characteristic Raman peaks belonging to α3ß1 integrin overexpressing EVs (Figure 16b). This study also showed the potential of developing a target‐specific SERS detection approach for characterizing EVs from a heterogeneous mixture in body fluids for cancer diagnosis. To achieve more robust tumor‐derived EV quantification, Zong et al. presented a SERS‐based detection method using magnetic nanoparticles as the EV capture substrate, and gold core–silver shell nanorods as SERS nanotags—both of which were functionalized with target‐specific antibodies (Figure 16c).[qv: 101d] The LOD was determined to be 1200 EVs (Figure 16d). Furthermore, the proposed method was simple and rapid (≈2 h) for direct detection of EVs from cell culture media without pre‐enrichment of EVs.

#### 3.2.2 Tumor‐Derived EV Phenotyping

Accurate EV phenotype profiling may facilitate the discovery of tumor‐specific EV subpopulations for cancer diagnosis. Kwizera et al. developed a miniaturized immunoaffinity‐based device combined with SERS readouts for the detection and protein profiling of tumor‐derived EVs.[qv: 101b] Gold nanorods coated with QSY21 Raman reporters were used as SERS nanotags. The SERS nanotag identified the captured EVs via the electrostatic interaction between positively charged gold nanorods (due to bilayer CTAB coatings) and negatively charged EVs (due to lipid membranes). This assay specifically detected EVs with an LOD of 2 × 10^6^ EVs mL^−1^. Using this assay, they found that EVs derived from diverse origins (i.e., different breast cancer cell lines and normal breast cells) possessed distinct protein profiles representative of their parental cells. The assay accuracy in characterizing protein profiles of EVs was further compared with ELISA, showing a correlation coefficient *R* ^2^ of 0.97. Furthermore, they applied the strategy for EV analyses in ten breast cancer patients. They concluded that the EV concentration was dependent on the individual irrespective of disease states, and EpCAM‐ and human epidermal growth factor receptor 2 (HER2)‐expressing EVs could potentially be used as biomarkers for diagnosis of HER2‐positive breast cancer patients.

**Figure 17:**
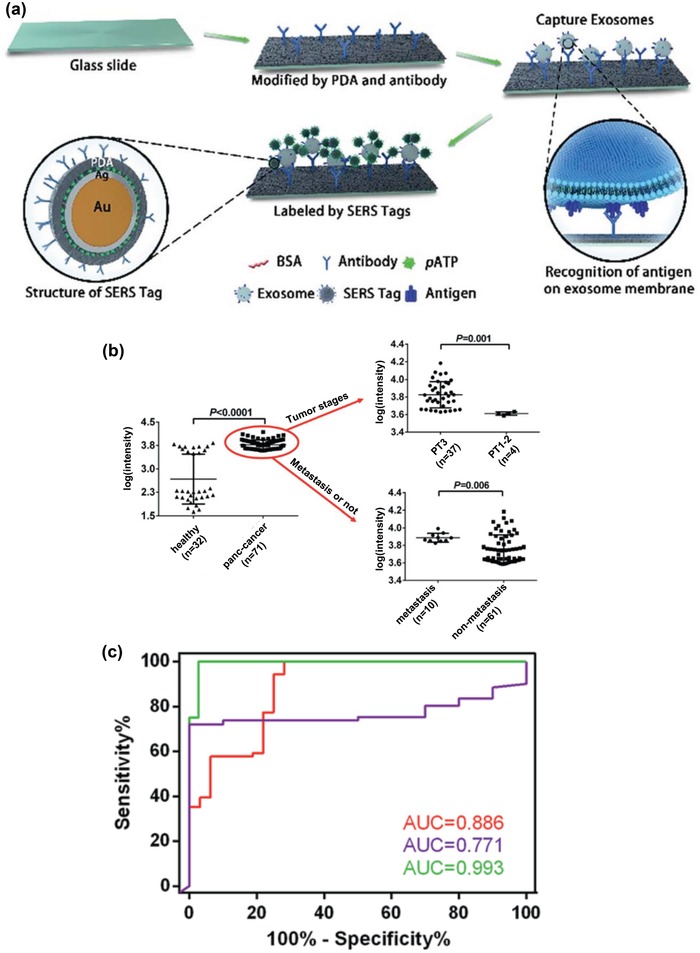
a) Schematic illustration of a SERS method that combines immunocapture‐based substrates and gold core–silver shell nanoparticles based SERS readouts for tumor‐derived EV detection. b) Shapiro–Wilk analysis plots of the SERS results of serum EV samples obtained from pancreatic cancer patients (*n* = 71) and healthy controls (*n* = 32) using the anti‐MIF platform. c) ROC curves were calculated (red: pancreatic cancer vs healthy controls; purple: metastasis vs nonmetastasis; and green: P1–2 versus P3 stages). AUC stands for area under the ROC curve. Reproduced with permission.[qv: 101c] Copyright 2018, The Royal Society of Chemistry.

In addition to cancer diagnosis, the identification of specific EV subpopulations can be applied to evaluate cancer metastasis stages. Li et al. developed a strategy that combined immunocapture‐based substrates and gold core–silver shell nanoparticle‐based SERS readouts for tumor‐derived EV detection (**Figure** **17**a).[qv: 101c] SERS nanotags and capture substrates were functionalized with antibodies for specific capture and identification of tumor‐derived EVs, with a low LOD of 1 EV in 2 µL of the sample solution. They further applied this technology for detecting tumor‐derived EVs with the expression of migration inhibitory factor (MIF) from pancreatic cancer patients (*n* = 71) and healthy individuals (*n* = 32) using 2 µL of the clinical serum sample. Shapiro–Wilk analysis plots (Figure 17b) and receiver operating characteristic (ROC) curve (Figure 17c) indicated that MIF‐expressing EVs might be a promising biomarker for differentiating patients from healthy individuals, metastasized tumors from metastasis‐free tumors, and Tumor Node Metastasis P1–2 stages from the P3 stage. This platform thus has great potential to be used for cancer diagnosis and staging.

#### 3.2.3 Outlooks for SERS‐Based Tumor‐Derived EV Analysis

To date, SERS‐based EV strategies including quantification and molecular characterization have increasingly been proposed and demonstrated for potential applications in cancer diagnosis, classification, and metastasis monitoring.[qv: 101c,102] Compared to conventional protein‐based EV detection methods (e.g., western blot: ≈10^10^ EVs mL^−1^, ≈10 h; ELISA: 10^8^ EVs mL^−1^, ≈5 h),[qv: 101c,103] SERS‐based EV strategies are more sensitive (≈1 EV per 2 µL sample solution) and quicker (≈2 h).[qv: 101d] Furthermore, SERS‐based EV strategies might shed light on EV heterogeneity in protein, lipid, and cytosolic composition by characterization of molecular fingerprint information of individual EVs,[qv: 100c] which is not achievable with either western blot/ELISA or PCR‐based methods. To ultimately boost the clinical translation of EVs, we thus provide the following suggestions. First, it is essential to select the most suitable isolation technology according to the purpose of investigation, downstream analyses to be performed, in addition to available equipment and resources. The purity of resulting EVs will affect the accuracy and performance of SERS analyses. For example, commercial purification kits (polymeric precipitation mixtures) can interfere with eventual Raman fingerprint spectra.[qv: 100b] Moreover, other less‐stringent purification protocols (i.e., differential ultracentrifugation and commercial precipitation kits) are subjected to limited purity due to co‐enrichment of protein aggregates and other nonvesicle materials, which might preclude SERS substrates from interacting with EVs, and interfere with eventual Raman fingerprint spectra. Second, it could be worth developing an ultrasensitive and specific SERS strategy for direct tumor‐derived EV detection from body fluids without the need for preisolation of EVs. This will significantly accelerate the EV analysis workflow and ultimately facilitate applications of EVs. Third, there is a need for extensive clinical evaluation based on a sizeable number of clinical samples, and comprehensive validation against a standard analysis method in order to move SERS‐based EV detection beyond the initial proof of concept.

### 3.3 Circulating Cancer Protein Biomarkers

A vast majority of circulating proteins have been discovered to be clinically useful for cancer diagnosis and disease monitoring.^104^ However, the protein component of body fluids is complex. For example, serum contains ≈10 000 proteins and is dominated by 22 high‐abundance proteins (e.g., albumin, immunoglobulin, and transferrin) that constitute 99% of the total protein mass of serum.^105^ The presence of these high‐abundance proteins could mask the low concentration of cancer‐specific proteins, leading to difficult detection.^106^ There is also ample evidence suggesting that a panel of protein biomarkers provide more information than a single one for clinical diagnosis,^107^ as the latter does not display sufficient discriminatory power to assist in clinical decisions. Several conventional approaches have been applied for serum protein analyses; such as western blot, reverse phase protein array, various antibody arrays, and ELISA. Western blot needs ≈10 h assay time and is not feasible to simultaneously detect proteins of similar molecular weights. Array‐based technologies generally rely on antibody‐functionalized platforms for specific capture of target proteins, followed by antibody‐tagged enzymes or fluorescent molecules for signal readouts. Nevertheless, such enzyme‐ or fluorescent‐based signaling methods are inherently subjected to either long assay time, limited sensitivity, or multiplexing capacity. For example, conventional ELISA needs ≈5 h assay time and is dependent on the spatial multiplexing mode for multiple protein biomarker detection. The detection sensitivity for western blot and ELISA is ≈pg. To advance the impact of proteomics in clinical use, it is thus essential to develop novel technologies that could provide sufficient sensitivity, specificity, reproducibility, and accuracy.

During the past decade, an increasing number of SERS‐based protein detection technologies have been proposed, showing excellent analytical sensitivity and multiplexing capacity. Generally, SERS‐based protein detection could be performed via i) target‐triggered nanoassembly assays; ii) 2D substrate‐based immunoassays;^108^ iii) pull‐down immunoassays;^39^, ^109^ and iv) microfluidic immunoassays.^110^ The captured proteins are then detected based on either the “fingerprint” information of target proteins^111^ or the characteristic spectra of Raman reporters conjugated to plasmonic nanomaterials.[qv: 24d,39,108–110,112] Although both SERS readout methods can provide sensitive and specific detection of circulating target proteins, nanotag‐based SERS immunoassays are more commonly used due to the availability of direct multiplex readouts based on unique spectral signals and strong signals provided by the Raman reporters with large SERS cross sections. We thus focus on the discussion of Raman reporter‐based SERS immunoassays in the ensuing subsection.

#### 3.3.1 Target‐Triggered Nanoassembly Assays

**Figure 18:**
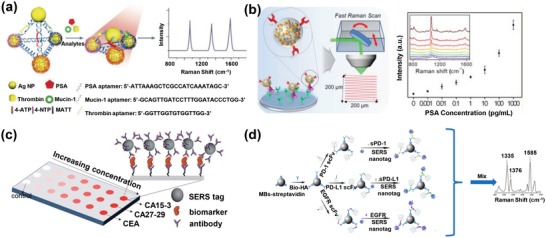
a) Illustration of silver pyramid self‐assembly by DNA frame as a SERS sensor for multiplex detection of cancer‐specific circulating protein biomarkers (PSA, thrombin, and Mucin‐1). Reproduced with permission.[qv: 24d] Copyright 2015, Wiley‐VCH. b) Schematic illustration of a 2D substrate‐based SERS immunoassay for PSA detection. Reproduced with permission.[qv: 108b] Copyright 2016, American Chemical Society. c) Schematic representation of a parallel 2D substrate‐based SERS immunoassay for the detection of multiple protein biomarkers (CEA, CA27‐29, and CA15‐3). Reproduced with permission.[qv: 108e] Copyright 2015, The Royal Society of Chemistry. d) Schematic illustration of using a pull‐down immunoassay based on the multiplex SERS readout for the detection of sPD‐1, sPD‐L1, and sEGFR. Reproduced with permission.^39^ Copyright 2018, American Chemical Society.

By using the binding affinity between proteins and specific‐target biomolecules (e.g., aptamer), protein detection has been done in the solution phase.[qv: 24d,112a,d,113] For example, Xu and co‐workers used silver pyramids which were self‐assembled by a DNA aptamer frame as a SERS sensor for simultaneous detection of cancer‐specific circulating protein biomarkers (prostate‐specific antigen (PSA), thrombin, and Mucin‐1). The sensor exhibited excellent sensitivity down to sub‐attomolar concentrations (**Figure** **18**a).[qv: 24d] The presence of target circulating proteins was identified by target‐specific aptamers that were embedded in the DNA frame. The resulting SERS signals increased in the presence of target proteins. Similarly, their colleagues synthesized bimetallic core–satellite nanostructures, and silver nanoparticle trimers for the detection of Mucin‐1 and α‐fetoprotein, respectively.[qv: 112a,d] These target‐triggered nanoassembly assays have demonstrated their advantages for rapid detection; however, the reproducibility might be poor due to uncontrollable aggregation states.

#### 3.3.2 2D Substrate‐Based Immunoassays

In most existing studies, circulating protein targets were captured by 2D substrates which were functionalized with target‐specific ligands before SERS detection. For such SERS immunoassays, 2D substrates in use generally include glass substrates,[qv: 108b] quartz slides,[qv: 108e] or gold substrates.[qv: 108a] Chang et al. developed a SERS immunoassay using an anti‐PSA functionalized glass slide with spot arrays for PSA capture,[qv: 108b] followed by SERS mapping readouts (Figure 18b). Each SERS nanotag comprised a silica core covered with 10 nm sized silver nanoparticles, and exhibited homogeneous signal intensities and single‐particle detection sensitivity. This platform offered the LOD of ≈0.11 pg mL^−1^ PSA and a wide dynamic range from 0.001 to 1000 ng mL^−1^. Compared to clinically used methods such as immunoradiometric assays and chemiluminescence immunoassays, this SERS method provided a better detection sensitivity and linearity at the detection of less than 0.1 ng mL^−1^ of PSA.

2D substrates with multiple wells have been developed for parallel SERS immunoassays involving multiple cancer protein biomarkers being detected in separate wells.[qv: 108a,e] Li et al. patterned multiple wells on a quartz substrate for the parallel SERS detection of breast cancer biomarkers, including cancer antigen CA15‐3, CA27‐29, and cancer embryonic antigen (CEA) (Figure 18c).[qv: 108e] They used gold nanostar SERS nanotags with a maximum LSPR peak at 748 nm (which is in resonance with the 785 nm excitation wavelength), and scattered radiation of Raman reporter 4‐nitrobenzenethiol at 877 nm to achieve sensitive SERS detection.^114^ The resultant LODs were 0.99 U mL^−1^, 0.13 U mL^−1^, and 0.05 ng mL^−1^ for CA15‐3, CA27‐29, and CEA in serum, respectively, which were superior to commercial ELISA kits. Recently, Banaei et al. applied a parallel SERS immunoassay for early diagnosis of pancreatic cancer based on protein biomarker profiles of CA19‐9, MMP7, and MUC4.[qv: 108a] In addition to designing SERS nanotags that possess a maximum LSPR band in resonance with excitation and scattered fields, they utilized a gold‐coated silicon substrate as the capture platform due to its higher signal enhancement as compared to glass or gold nanopillar substrates. The gaps between gold‐coated silicon substrates and gold nanoshell SERS nanotags also served as plasmonic nanogaps for additional signal enhancement. They demonstrated that their SERS immunoassay was sensitive to 2 ng mL^−1^ of each target protein biomarker in buffer solution. They further measured the levels of these biomarkers in pancreatic patients (*n* = 5), pancreatitis patients (*n* = 5), and healthy individuals (*n* = 5). They observed a unique expression pattern of these biomarkers in individuals, and relatively higher expression levels of each biomarker in pancreatic patients as compared to the other groups. According to the *Z*‐score of each biomarker, they determined CA19‐9 to be the best predicting biomarker for differentiating pancreatic and pancreatitis patients. Despite the high detection sensitivity, 2D substrate‐based SERS protein detection is generally time‐consuming as at least 3 h incubation time is required for efficient capture and labeling of target proteins.[qv: 108a,b] Additionally, the above‐mentioned parallel SERS detection methodologies have not demonstrated “single‐tube” detection of multiple target proteins, and might cause unexpected variations in measuring the relative expression levels of protein biomarkers due to different measurement conditions, and additional consumption of precious limiting samples.

Tang et al. developed a multiplex SERS frequency shift immunoassay for the simultaneous detection of liver cancer protein biomarkers captured by an antibody‐ and Raman reporter‐functionalized 2D substrate.[qv: 112c] The bifunctionality of this 2D substrate was achieved by directly conjugating capture antibodies to Raman reporters which were bound to the surface of silver nanoparticle–coated glass slides. The SERS signal readout was based on spectral shifts of Raman reporters upon target circulating protein capture. This multiplex SERS frequency shift immunoassay was sensitive in the detection of two different target proteins at sub‐picomolar concentrations. As compared to the common “sandwich” structure–based immunoassay that involves at least two steps of immunoreaction, this strategy is faster as only a single immunoreaction step is required for the detection of multiple target proteins. Nevertheless, the multiplexing potential of this strategy is subjected to the availability of Raman reporters that can sense the “nanostress,” and transduce into frequency shifts of highly resolved Raman peaks.

#### 3.3.3 Pull‐Down Immunoassays

Pull‐down SERS immunoassays involve protein isolation by adsorbing protein complexes onto beads. Pull‐down immunoassays allow for multiplex detection in one pot with universal Raman reporters, and are relatively faster than immunoassays on 2D substrates. Cheng et al. developed a pull‐down immunoassay using gold nanosphere SERS nanotags to determine the ratio of free to total PSA (including free and complexed PSA) with the aim of improving the diagnostic performance of prostate cancer.[qv: 109a] The LODs of free PSA and complexed PSA measured by this duplex pull‐down immunoassay were estimated to be 0.012 and 0.15 ng mL^−1^, respectively. Importantly, this assay platform only required less than 10 µL of serum for each assay and less than 1 h experimental time. The authors also demonstrated that the duplex measurement of free to total PSA provided a better precision than the parallel assay (the coefficient variations of 6.57% vs 16.38%). This methodology was further applied for the detection of dual PSA biomarkers in 13 clinical samples and compared against the standard electro‐chemiluminescence assay to show clinically acceptable consistency.

The detection sensitivity and linear detection range of pull‐down immunoassays could be further improved by using advanced SERS substrates. He et al. demonstrated an ultrasensitive pull‐down immunoassay of detecting N‐terminal probrain natriuretic peptide by using metal–organic frameworks embedded with gold tetrapods as SERS substrates.[qv: 109b] This type of SERS substrate possessed excellent characteristics such as high porosity, large surface area, and good stability, which enhanced SERS signaling dramatically. The proposed SERS immunosensor achieved a large dynamic range of six orders of magnitude from 1 fg mL^−1^ to 1 ng mL^−1^ with an LOD of 0.75 fg mL^−1^. Meanwhile, Li et al. have employed surfactant‐free gold‐silver alloy nanoboxes as SERS substrates for simultaneous detection of a panel of magnetic particle–captured soluble cancer protein biomarkers (Figure 18d).^39^ They successfully detected soluble programmed death 1 (sPD‐1), programmed death‐ligand 1 (sPDL‐1), and epithermal growth factor receptor (sEGFR) with LODs of 6.17, 0.68, and 69.86 pg mL^−1^, respectively. This SERS technique achieved detection sensitivity equal to or lower than ELISAs (pg mL^−1^ to ng mL^−1^) with simultaneous multiplexing detection capability. In conclusion, pull‐down immunoassays have been demonstrated to be a fast and sensitive immunosensor.

#### 3.3.4 Microfluidic Immunoassays

For both immunoassays on 2D substrates and pull‐down immunoassays, the interaction between antibody‐functionalized substrates/particles and target proteins mainly relies on random movement (e.g., Brownian motion). Microfluidic devices that incorporate on‐chip active mixing have recently been proposed to speed up the detection workflow by increasing diffusion kinetics and binding efficiencies. Additionally, microfluidic devices also attract tremendous interest for precise control of fluids, low consumption of samples and reagents, and streamlined sample processing.

**Figure 19:**
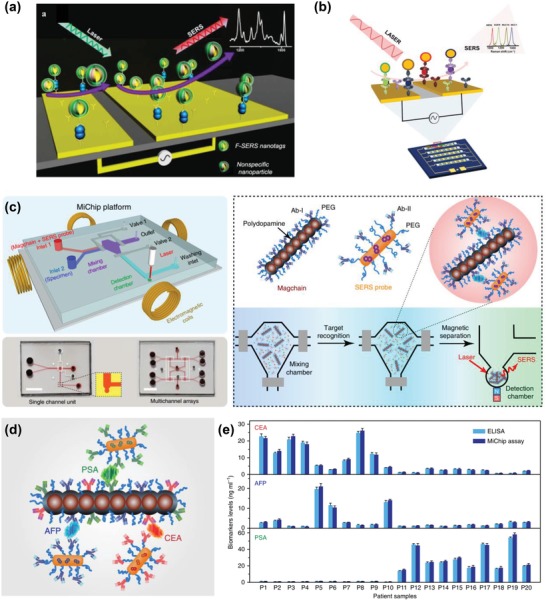
a) Scheme of ac‐EHD‐induced microfluidic‐SERS immunoassay. Reproduced with permission.[qv: 110c] Copyright 2015, American Chemical Society. b) Representation of ac‐EHD‐induced microfluidic‐multiplex SERS immunoassay. Reproduced with permission.[qv: 110a] Copyright 2017, Wiley‐VCH. c) Design of a magnetic nanochain–integrated microfluidic chip for the detection of cancer protein biomarkers. d) Conceptual illustration of multiplex detection of cancer protein biomarkers. e) Comparison of protein biomarker levels in 20 clinical serum specimens measured by the magnetic nanochain–integrated microfluidic chip and ELISA. Reproduced with permission.[qv: 110d] Copyright 2018, Nature Publishing Group.

Wang et al. have utilized alternative current electro‐hydrodynamic (ac‐EHD)‐induced nanoscaled surface shear forces to enhance target capture kinetics, and gold/silver nanoshells as SERS nanotags (**Figure** **19**a).[qv: 110c] The nanoscaled physical forces acting within nanometer distance from the electrode surface enabled rapid (40 min), sensitive (10 fg mL^−1^), and highly specific detection of HER2 in serum samples of breast cancer patients. More importantly, this ac‐EHD‐induced microfluidic‐SERS platform has been demonstrated to significantly reduce nonspecific binding events caused by the use of polyclonal antibodies.^115^ The reduction of nonspecific binding is of great importance for the analysis of complex real body fluid samples. Given these advantages of the ac‐EHD‐induced microfluidic‐SERS platform, Reza et al. have further extended this platform for extensive multiplex protein sensing, and showed high detection sensitivity for each protein biomarker at 10 fg mL^−1^ in serum samples (Figure 19b).[qv: 110a,b]

Recently, Xiong et al. reported a microfluidic‐SERS platform that is comprised of a magnetic nanochain integrated microfluidic chip and a multiplex SERS nanotag system for the rapid and simultaneous detection (<8 min) of multiple cancer protein biomarkers from small volumes (1 µL) of serum samples (Figure 19c,d).[qv: 110d] The magnetic nanochain–integrated microfluidic chip was built upon the synergistic utilities of nanochains as nanoscale stir bars for rapid liquid mixing, and as capturing agents for specific bioseparation. This platform provided a linear signal response ranging from 0 to 100 ng mL^−1^ for the detection of three cancer protein biomarkers from simulated patient serum samples. This platform has been further used to test 20 patient samples, showing good agreement with ELISA results (Figure 19e). Taken together, the advanced microfluidic SERS platform significantly accelerates the analysis of circulating cancer proteins with high sensitivity and specificity.

#### 3.3.5 Outlooks for SERS‐Based Circulating Cancer Protein Biomarker Analysis

There are various types of SERS‐based protein detection platforms developed for disease diagnosis. Generally, these SERS platforms can achieve the LOD ranging from fg mL^−1^ to pg mL^−1^ and at least simultaneous 4‐plex detection.[qv: 110a] The integration of microfluidic devices has further improved detection specificity and reduced the assay time down to ≈8 min.[qv: 110c,d] SERS‐based protein detection platforms thus possess great advantages compared to conventional protein assays such as western blot and ELISA in terms of improving specificity and reducing assay time. Currently, these aforementioned advanced SERS–protein assays have only been applied for the discrimination of cancer patients and healthy individuals based on the concentration of circulating cancer protein biomarkers. It may be worth exploring the potential of SERS–protein assays in broader clinical applications, such as cancer subtyping and treatment monitoring. Furthermore, it might be of interest to put in effort on the commercialization of SERS‐based protein assays, which requires i) precise quantification capability; ii) negligible interference from other components in real samples; and iii) miniaturized, portable, and low‐cost platforms.

### 3.4 Circulating Tumor Nucleic Acids

The potential origins of NAs in body fluids include CTCs, stem cells, blood cells, and viruses. These origins release NAs into circulation through passive mechanisms such as apoptosis and necrosis, or active mechanisms such as spontaneous secretion from cells. Typically, circulating cancer‐specific NAs include circulating tumor DNA (ctDNA), circulating DNA of viruses, microRNA (miRNA), and gene fusion RNA. The genetic profiling of these circulating cancer‐specific NAs can further complement with tumor biological information obtained through the analysis of CTCs, tumor‐derived EVs, and circulating cancer proteins. For example, ctDNAs have a short half‐life (<2.5 h) and thus can represent more timely, dynamic information of tumor progression than other biomarkers.^116^ Additionally, the concentration of ctDNA in plasma has been shown to correlate with tumor size^117^ and stage,^118^ although a substantial variation in ctDNA concentration may exist between individuals. Furthermore, ctDNA has been reported to be detectable in early‐stage cancer patients.^118^, ^119^ miRNAs are also promising biomarkers given the difference in the concentration and composition of miRNAs between cancer patients and healthy individuals.^120^ The analysis of these circulating tumor NAs can be performed with established techniques such as quantitative PCR (qPCR), droplet digital PCR, and NGS. These technologies largely utilize fluorescence‐based readouts, and are consequently subjected to either expensive reagents, limited sensitivity (whole genomic sequencing methods: >1–5% of circulating tumor NAs),^121^ or multiplexing capacity (e.g., ≈4 multiplexing capacity for qPCR).^122^ Due to the low abundance of circulating tumor NAs, it is desirable to develop novel approaches to address the limitations of current gold standard techniques.

#### 3.4.1 Direct Detection

To improve detection sensitivity and to reduce reagent costs, direct SERS detection of NAs on the surface of plasmonic nanomaterials without using any fluorescence‐labeled probes has been proposed. For example, Koo et al. developed a gold nanoparticle–based SERS system to noninvasively detect a cancer gene fusion RNA biomarker in urine after isothermal reverse transcription‐recombinase polymerization amplification (RT‐RPA) of targets.^123^ The gene fusion RNA biomarker (i.e., *TMPRSS2‐ERG*) in prostate cancer was specifically amplified with biotin‐deoxyuridine triphosphate incorporation, and then conjugated onto the surface of streptavidin‐coated gold nanoparticles for SERS measurements. This method was sensitive to 1000 copies of *TMPRSS2‐ERG* and has shown successful detection in urine samples of prostate cancer patients.

**Figure 20:**
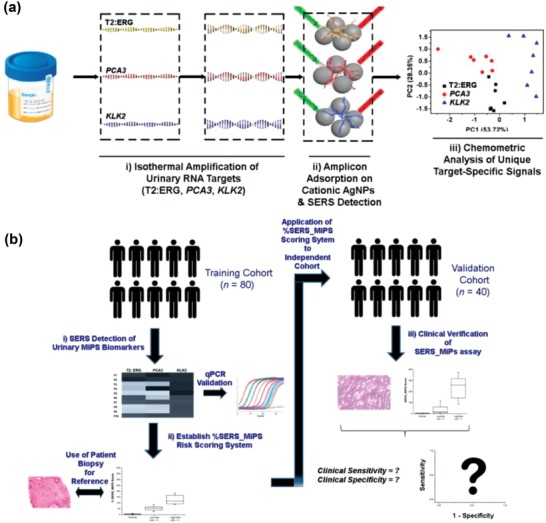
a) Scheme of direct SERS detection of urinary RNA targets. b) The clinical verification of direct SERS detection for individual prostate cancer risk prediction using a clinically relevant biomarker model, and two independent sample cohorts. Reproduced with permission.[qv: 124a] Copyright 2018, American Chemical Society.

Koo et al. have then shown an improved detection sensitivity of *TMPRSS2‐ERG* (i.e.,100 copies) by utilizing positively charged silver nanoparticles, which allowed for a favorable physical interaction between target sequences and SERS substrate surfaces for intensified SERS effect (**Figure** **20**a).^124^ Furthermore, they applied this method to differentiate *TMPRSS2‐ERG* and housekeeping *RN7SL1* RNA in 43 noninvasive patient urine samples based on the unique NA base composition of each sequence. The good resultant discriminatory sensitivity (95.3%) and specificity (93.0%) indicated the potential of using this technology for cancer genetic subtyping. Recently, they have progressed this method further toward clinical applications by comprehensively evaluating its clinical performance (Figure 20b). They investigated the clinical performance on independent training (*n* = 80) and validation (*n* = 40) cohorts of prostate cancer patient samples. They established a prostate cancer risk scoring system by correlating SERS outcomes to gold‐standard biopsy results, which exhibited a clinical sensitivity of 87% and a specificity of 90%. They also achieved an area‐under‐ROC curve value of 0.84 for differentiating high‐ and low‐risk prostate cancers.

#### 3.4.2 Nanotag‐Based Detection

**Figure 21:**
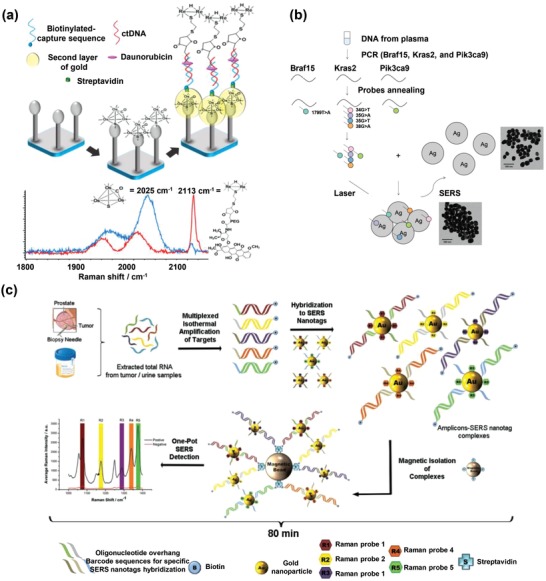
a) Scheme of using biointerference‐free probes for SERS detection of ctDNA of Epstein–Barr viruses in the blood. Reproduced with permission.^127^ Copyright 2018, American Chemical Society. b) Schematic of the PCR–SERS method for the detection of DNA mutations in plasma. Reproduced with permission.^128^ Copyright 2018, Ivyspring. c) Scheme of pentaplex RT‐RPA–SERS detection of RNA biomarkers in tumor and urine samples of prostate cancer patients. Reproduced with permission.^130^ Copyright 2016, Wiley‐VCH.

The use of SERS nanotags has enabled sensitive detection of circulating tumor NAs without any amplification process. Zhou et al. developed a DNA‐mediated SERS sensor coupled with RNase HII‐assisted amplification for the detection of ctDNA in blood.^125^ They designed a recognition unit for the specific identification of point mutations in *KRAS G12DM*, which is a diagnostic biomarker for colon carcinoma. In presence of *KRAS G12DM*, the recognition unit released signal transduction probes (i.e., thymine‐rich single‐stranded DNA) to facilitate in situ growth of copper nanoparticles on the surface of single‐walled carbon nanotubes, hence resulting in obvious signal enhancement at 1605 cm^−1^ peak of the G‐band. Through utilizing this efficient SERS signal enhancement mechanism, this strategy was able to detect as low as 0.3 × 10^−15^ m of *KRAS G12DM* in human blood and has been demonstrated for six patient samples. Similarly, Zheng et al. proposed a strategy that incorporated miRNA‐triggered hybridization chain reaction and silver iron–mediated SERS signal amplification, which was capable of detecting as low as 0.3 × 10^−15^ m of miRNA in the buffer.^126^ Recently, Lin et al. demonstrated a combination of SERS‐active nanopillar substrates and biointerference‐free probes (i.e., rhenium carbonyl) for improving the detection sensitivity of circulating DNA by reducing signal interference from nontarget DNA and biomolecules in plasma (**Figure** **21**a).^127^ This strategy was sensitive for the detection of 500–1000 copies mL^−1^ of cancer‐associated Epstein–Barr virus DNA in blood circulation. The potential feasibility of this strategy was tested using 15 cancer patient samples, showing higher expression of the cancer‐specific DNA in patient blood as compared to normal samples. Furthermore, the obtained results were in good agreement with PCR data, suggesting the potential of this technique in clinical applications.

The detection sensitivity of circulating tumor NAs could be further improved via the integration of amplification methods and probe‐based SERS readouts. Li et al. proposed a PCR–SERS method to simultaneously detect multiple cancer gene mutations (i.e., *BRAF*, *KRAS*, and *PIK3CA*) in plasma of colorectal cancer patients (Figure 21b).^128^ The PCR amplicons were tagged with fluorescence‐labeled probe sequences that were complementary to the target mutations. After removing unbound probes via a purification process, the tagged probes were then extracted and incubated with silver nanoparticles for SERS measurements. The LOD was determined to be 5.15 × 10^−11^ m. This method was further applied to investigate the frequencies of targeted mutations (*V600E*, *G12C*, *G12D*, *G12V*, *G13D*, and *E542K*) in 49 patient plasma samples. The results indicated a higher frequency of *BRAF‐V600E* and *PIK3CA‐E542K* mutations in right‐sided colon cancer patients than left‐sided colon and rectal cancer patients. Although this PCR–SERS method can easily leverage existing PCR conditions, the multiplex target detection still required statistical methods to spectrally deconvolute the SERS signals of fluorescence‐labeled probes.

To enable a simple signal readout in a multiplex target detection system, SERS nanotags with minimal signature overlaps were employed. For example, Wee et al. incorporated the standard PCR assay with a multiplex SERS barcoding system for ultrasensitive detection of cancer DNA mutations (*BRAF V600E*, *c‐Kit L576P*, and *NRAS Q61K*) in ctDNA from serum samples of melanoma patients.^129^ Basically, generated PCR amplicons were tagged with biotin molecules and target‐specific overhang hybridization sequences. Different amplicons were then labeled with respective SERS nanotags through complementary sequence hybridization for SERS readouts. The enrichment of SERS nanotag‐labeled PCR amplicons and simultaneous removal of unbound SERS nanotags were then achieved by streptavidin‐coated magnetic beads. This proof‐of‐concept method reproducibly detected as few as 10 mutant alleles from the background of 10 000 wild‐type sequences (0.1%; coefficient of variability < 9%). They further applied this method to detect DNA mutations in cell lines and serum where results were subsequently validated with droplet digital PCR. Similarly, the multiplex SERS readout could also be incorporated with RT‐RPA for the sensitive detection (100 copies) of noninvasive RNA biomarkers from tumor and urine (Figure 21c).^130^ As RT‐RPA is an isothermal amplification technique, the use of RT‐RPA significantly sped up the amplification process, and thus offered 80 min subtyping of a prostate cancer tumor or a urine sample based on 5‐plex RNA biomarker profiles. The obtained results were further validated with standard quantitative reverse transcription‐PCR (qRT‐PCR) with 100% concordance.

#### 3.4.3 Outlooks for SERS‐Based Circulating Tumor NA Analysis

All the above‐mentioned SERS technologies have demonstrated excellent sensitivity and accuracy in the detection of circulating tumor NAs in patient body fluids for cancer molecular subtyping and risk prediction.[qv: 124a,129,130] The detection sensitivity of mutant alleles using SERS has been shown to be 0.1%,^129^ which is tenfold higher than that of commercial PCR‐based assays (1%).^131^ Additionally, the multiplexing capacity provided by the reported nanotag‐based SERS strategy is 6, and may be potentially increased by introducing different SERS nanotags. The combination of RT‐RPA and SERS has enabled 5‐plex detection in a shorter assay timeframe (80 min) than conventional techniques such as qRT‐PCR and NGS (>2.5 h from the sample to answer). However, to facilitate the clinical translation of SERS‐based circulating tumor NA detection, it is essential to comprehensively evaluate their clinical performance using a large number of patient samples. It might also be worth exploring the feasibility of SERS‐based circulating tumor NA detection in addressing specific clinical applications, such as early cancer detection or treatment monitoring. Also, refinement of assay workflows could promote the practical applications of some SERS‐based circulating tumor NA detection strategies. For example, the biointerference‐free probe‐based SERS detection requires multiple labeling and detection steps,^127^ which needs to be further simplified to improve detection efficiency.

## 4 Summary and Perspectives

State‐of‐the‐art plasmonic nanomaterials have found prosperous applications in the field of liquid biopsy analyses. In this review, we provide an overview of the current state of engineered plasmonic nanomaterials, and how they are integrated into SERS platforms for liquid biopsy analyses. It is to be noted that considerable progress has been achieved in both development of high‐performance plasmonic nanomaterials, as well as their diverse demonstrated applications in SERS‐based liquid biopsy analyses. Meanwhile, significant efforts are being invested in improving overall plasmonic nanomaterial–based SERS performance for clinical liquid biopsy applications. For example, Li et al. built a gold–areole array on patterned superhydrophilic–superhydrophobic substrates to concentrate analytes in hot spot zooms.^132^ Li et al. developed a 3D aluminum hybrid nanostructure with a high density of hot spots across a large scale (≈1 cm^2^),^133^ and the protective alumina layer further provided long‐term stability of plasmonic properties and SERS performance (≥6 months). Despite these great advances, there are still several roadblocks to overcome before successful deployment of clinical SERS‐based liquid biopsy platforms for genuine patient benefit. To end off, we provide below our insights and possible directions in achieving this lofty goal.

1) *Improve the Colloidal Stability of SERS Nanotags in Biological Systems*: In recent research, Wang et al. have demonstrated that the reproducibility and accuracy of nanotag‐based SERS detection rely on the colloidal stability of SERS nanotags.^134^ They found that the universal practice for particle aggregation prevention by coating SERS nanotags with silica or bovine serum albumin layers did not sufficiently stabilize particles in biofluid environments such as blood. We thus strongly recommend to i) further improve the colloidal stability of SERS nanotags; and ii) characterize particle stability in the measurement environments that particles will be used in or include proper controls to normalize aggregation artifacts.
2) *Explore Alternative Nanomaterials for SERS‐Based Liquid Biopsy Applications*: In addition to plasmonic nanomaterials, other materials such as plasmonic‐free semiconductors,^135^ 2D materials,^136^ and hybrid plasmonic/2D materials^137^ have emerged as high‐performance SERS‐active substrates. These modernistic nanomaterials provide new possibilities for SERS detection due to their unique electronic and optical properties, together with good chemical stability and biocompatibility. However, only a few of these alternative nanomaterials have been applied in SERS biosensing platforms for liquid biopsy analyses. The first potential reason is that scientists working on biosensor development for liquid biopsy analyses may have limited knowledge of advanced SERS‐active substrate synthesis, and are unaware of the latest developments of these nanomaterials. The other possible reason is that the reproducibility of nanomaterial generation is often poor due to the usage of chemicals from different companies, different experimental environments (e.g., air temperature, air humidity, and particulate matter in the air), and insufficient key information in the published synthesis protocol. As such, it is important to standardize the synthesis protocol, as well as strengthen the collaboration among the nanomaterial synthesis, SERS application, and end‐user clinical communities to facilitate the applications of alternative plasmonic nanomaterials for liquid biopsy analyses.
3) *Facilitate the Development of User‐Friendly SERS Liquid Biopsy Sensing Systems*: Currently, there are many simple and rapid SERS sensing platforms which have been developed for liquid biopsy analyses.[qv: 93,110d,124a] Still, these sensing strategies need to be performed by well‐trained technicians with applicable laboratory and data analysis skills (e.g., pipetting, Raman measurement, and statistical analysis). To promote the translation of SERS liquid biopsy sensing systems from lab to practical clinical usage, it could be beneficial to i) design an automated workflow for liquid biopsy preparation to signal transduction; ii) develop portable Raman spectrometers for convenient sample detection; and iii) integrate a software program with signal measurements, data processing, and result analyses to simplify detection outcome interpretation. Besides user‐friendliness, clinical assay development should also consider practical issues, such as assay costing, that determine eventual acceptance by the healthcare community.
4) *Perform a Comprehensive Clinical Evaluation of SERS‐Based Platforms*: Robust analytical performance is an essential but inadequate prerequisite for the successful clinical deployment of SERS liquid biopsy sensing platforms. Currently, most SERS liquid biopsy sensing platforms still rely on the use of simulated or limited patient samples to assess proof‐of‐principle feasibility and analytical performance, without accounting for biological complexities and variabilities of human patient samples. Furthermore, only a very few sensing platforms have been designed for addressing specific clinical challenges such as early‐cancer detection or treatment monitoring disease. As such, to expedite clinical translation, we believe that these SERS liquid biopsy sensing systems need to involve i) clinical purpose‐oriented experimental design; ii) testing on a statistically relevant cohort of relevant clinical samples; and iii) comparisons against current established gold standard analysis techniques. Typically, parameters to evaluate the clinical performance should include clinical sensitivity, clinical specificity, and diagnostic accuracy. To translate SERS liquid biopsy sensing platforms in clinical practice, a further demonstration of their clinical validity, clinical utility, and economic evaluation is also needed.[qv: 124a,138]

## Conflict of Interest

The authors declare no conflict of interest.
